# SARS-CoV-2 Omicron infection reveals imprinted antibody responses in the absence of vaccination

**DOI:** 10.1016/j.isci.2026.115910

**Published:** 2026-04-28

**Authors:** Adam Abdullahi, Rebecca B. Morse, Mark Tsz Kin Cheng, Fehintola Ige, James Onyemata, Martin Edun, Anezka Kramna, Benjamin Sievers, Sam Turner, Haruna Wisso, Emmanuel Jonathan, Olasinbo Balogun, Abideen Salako, Hafsat Abdulazeez-Oha, Ayorinde James, Adesola Musa, Onisile Oluwaseun, Bamidele Iwalokun, Oliver Ezechi, Abbas Anzaku, Evaezi Okpokoro, Sophia Osawe, Gospel Nwikue, Ibrahim M. Kida, Baba Maiyaki Musa, Hassan Adam Murtala, Bo Meng, Leah Rosenzweig, Sani H. Aliyu, Derek Smith, Paul MacAry, Rainer Doffinger, Witold Więcek, Babatunde Salako, Chee Wah Tan, Alash’le Abimiku, Ravindra K. Gupta

**Affiliations:** 1 Cambridge Institute of Therapeutic Immunology & Infectious Disease (CITIID), Department of Medicine, University of Cambridge, Cambridge, UK; 2 International Research Centre of Excellence, Institute of Human Virology, Abuja, Nigeria; 3 Takemi Program in International Health, Harvard T.H Chan School of Public Health, Boston, MA, USA; 4 Nigerian Institute of Medical Research, Yaba, Lagos, Nigeria; 5 Center for Pathogen Evolution, Department of Zoology, University of Cambridge, Cambridge, UK; 6 Department of Pharmacology, PAMO University of Medical Sciences, Port Harcourt, Rivers State, Nigeria; 7 Department of Infectious Diseases and Clinical Immunology, University of Maiduguri Teaching Hospital, Maiduguri, Nigeria; 8 Department of Medicine, Bayero University Kano, Kano, Kano State, Nigeria; 9 Addenbrooke’s Hospital, Cambridge University Hospitals NHS Foundation Trust, Cambridge Biomedical Campus, Cambridge, UK; 10 Development Innovation Lab, University of Chicago, Chicago, IL, USA; 11 National University of Singapore, Singapore, Singapore; 12 College of Medicine, University of Ibadan, Ibadan, Nigeria; 13 Africa Health Research Institute, Durban, South Africa; 14 The Hong Kong Jockey Club Global Health Institute (HKJCGHI), Hong Kong SAR, China

**Keywords:** immunology, virology

## Abstract

Distinct from vaccine-first models, infection-first exposures provide a critical context for understanding SARS-CoV-2 immune imprinting in unvaccinated populations. We analyzed neutralizing antibody responses in two independent unvaccinated Nigerian cohorts sampled in early 2023. Using a BA.1 receptor-binding domain (RBD)-based assay for Omicron exposure discrimination, we identified widespread pre-Omicron and partial Omicron exposure. Despite recent Omicron infection, plasma neutralization titers against Omicron lineages remained equal or lower compared to those against ancestral Wu-1, indicating infection-derived imprinting. Depletion of Wu-1 spike-binding antibodies abrogated neutralization of both Wu-1 and Omicron pseudoviruses, confirming dominance of ancestral cross-reactive antibodies. Following Wu-1-based vaccination, neutralizing responses increased across all variants, yet Omicron titers did not exceed Wu-1 titers even after breakthrough infection. These findings demonstrate durable infection-induced immune imprinting established before vaccination and only partially mitigated by repeated Omicron exposures, underscoring the influence of infection-first exposure sequence on antibody breadth and broader global relevance for vaccine design.

## Highlights

• BA.1 RBD-Omicron serology discriminates between Omicron and Wu-1 exposure
• Unvaccinated Nigerian cohorts show strong Wu-1 imprinting despite Omicron infection
• Wu-1-spike antibody depletion abolishes Omicron neutralization
• RBD imprinting persists, only partially mitigated by Wu-1 vaccination + Omicron BTI

## Graphical abstract

**Figure:**
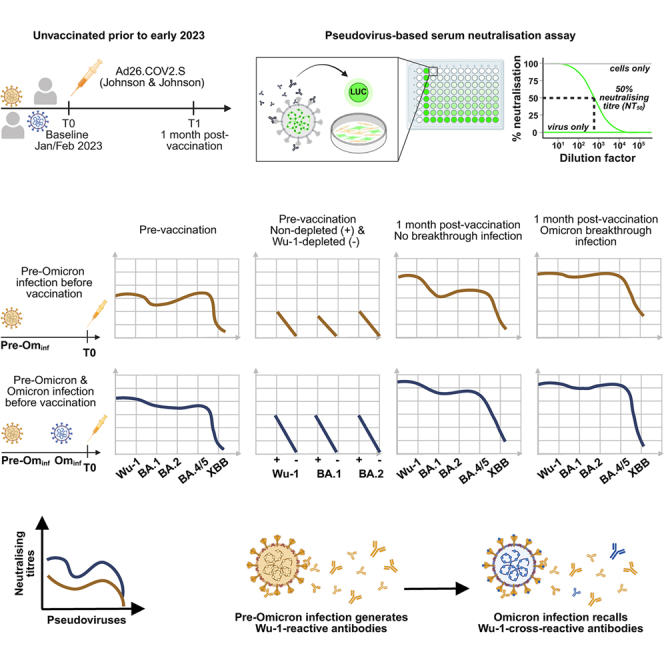

## Introduction

Despite widespread immunity from infection and vaccination, immune-evasive SARS-CoV-2 variants, often arising in immunocompromised hosts, continue to emerge and spread globally.^1^^,^^2^^,^^3^^,^^4^^,^^5^^,^^6^ Although vaccination remains the most effective long-term strategy for protection,^7^^,^^8^^,^^9^^,^^10^ sustained viral circulation has produced successive variants with altered antigenic landscapes.^11^^,^^12^ The emergence of Omicron (B.1.1.529) represented a radical antigenic shift, characterized by multiple extensive spike mutations that conferred extreme immune evasion and enhanced transmissibility.^13^^,^^14^^,^^15^^,^^16^^,^^17^^,^^18^^,^^19^^,^^20^

Immunological imprinting—a phenomenon where primary exposure to a pathogen shapes subsequent recognition of antigenically divergent strains is now recognized as a key determinant of SARS-CoV-2 antibody responses.^21^^,^^22^^,^^23^ It favors recall of existing memory B cell responses rather than *de novo* responses to novel epitopes and thereby limits the potency and breadth of humoral responses.^24^^,^^25^^,^^26^^,^^27^ Immune imprinting has been well demonstrated in individuals primed with ancestral Wu-1 vaccines and later exposed to Omicron. The imprinting effect manifests as Wu-1-skewed neutralizing antibodies despite recent Omicron exposure, driven by recall of pre-existing memory B cells limited *de novo* responses to Omicron,^8^^,^^28^^,^^29^^,^^30^^,^^31^^,^^32^^,^^33^^,^^34^ highlighting the dominant effect of prior antigenic exposure.^35^ Studies using antibody-depletion experiments have demonstrated that abrogation of Wu-1 spike-binding antibodies markedly reduces cross-neutralization, particularly in individuals without prior Omicron exposure, highlighting restricted breadth imposed by ancestral imprinting.^35^^,^^36^ This effect persists even after vaccination with updated Omicron-targeting vaccines, such as XBB.1.5 formulations, which continues to elicit responses dominated by recalled ancestral memory B cells.^36^ Moreover, ancestral imprinted responses persist despite multiple Omicron infections^37^ highlighting its durability across repeated exposures.

To date, most SARS-CoV-2 imprinting studies largely begin with vaccine-first exposures, where the ancestral Wu-1-based vaccine represented the primary antigenic encounter^34^^,^^38^^,^^39^^,^^40^^,^^41^^,^^42^^,^^43^^,^^44^^,^^45^ before Omicron exposure. The nature of the first exposure, however, critically shapes the resulting immune landscape. Infection tends to broaden epitope recognition^31^^,^^35^^,^^36^^,^^37^^,^^38^^,^^39^^,^^40^^,^^41^^,^^42^—targeting S2 and NTD domains and induces stronger B cell clonal expansion and higher somatic hypermutation rates,^46^^,^^47^ whereas vaccination predominantly recalls RBD-targeting antibodies.^40^ These mechanistic contrasts highlight the immunological importance of exposure sequence and raises the question whether individuals who experience Omicron infection before vaccination also develop imprinting. This question is particularly relevant for the African continent where limited early vaccine access meant that many individuals first encountered SARS-CoV-2^48^ via natural infection, including with Omicron-lineage viruses, before vaccination. With only about one-third of the African population completing a two-dose vaccination series by late 2023,^49^ such infection-first exposures provide a natural experiment to examine how imprinting develops without prior vaccine priming.

Here, we leveraged an unvaccinated population sampled during Nigeria’s primary vaccine rollout in early 2023 to validate a BA.1-RBD-based serological assay capable of distinguishing Omicron from pre-Omicron exposure. Thereafter, we demonstrate in two independent Nigerian cohorts that despite recent Omicron exposure, Omicron neutralizing antibody responses are paradoxically lower or equal to Wu-1 responses. Spike-specific antibody depletion further demonstrated that removal of Wu-1-binding antibodies abrogated neutralization of both BA.1 and BA.2, confirming infection-derived imprinting. Finally, we measure neutralizing antibody titers after Wu-1-based vaccination and demonstrate that imprinting by ancestral pre-Omicron variants is only partially mitigated by experiencing Omicron exposures through both pre-vaccination Omicron infection and post-vaccination breakthrough infection.

## Results

### Study population characteristics and SARS-CoV-2 exposure

**Figure 1:**
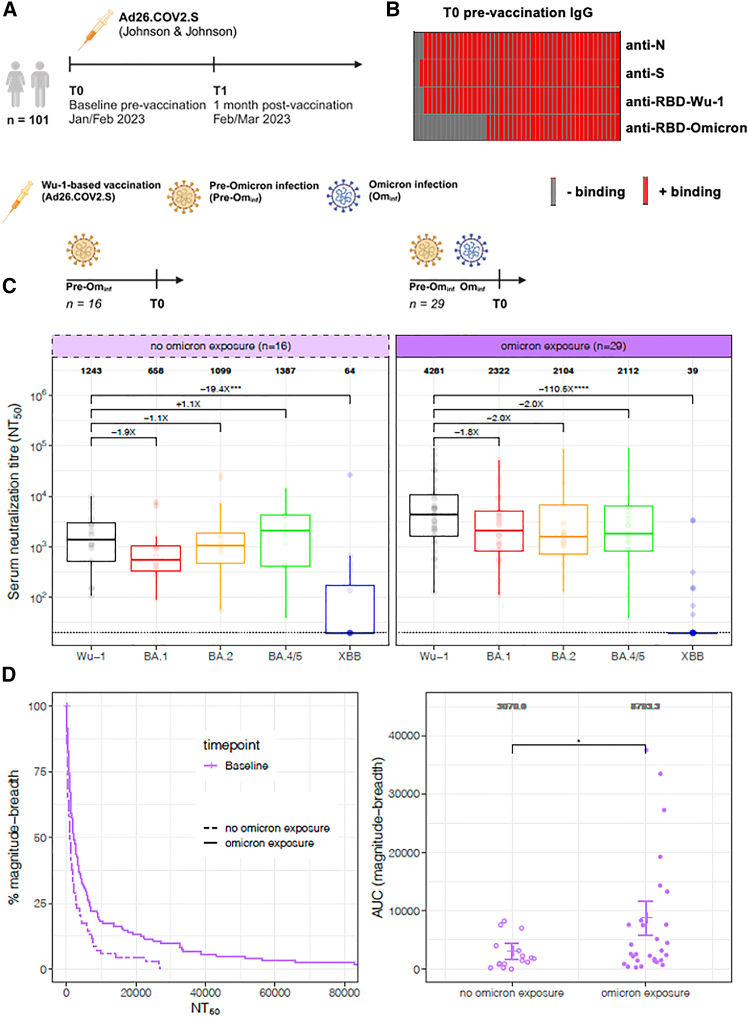
Binding antibody responses distinguish prior exposure to pre-Omicron versus Omicron variants (A) Schematic of cohort 1 study population. (B) Proportion of (*n* = 45) participants recruited with neutralization response data prior to vaccination (T0) who were tested for total IgG antibodies against SARS-CoV-2 wild-type (Wu-1) anti-nucleocapsid (anti-N), anti-spike (S), anti-Wu-1 receptor binding domain (anti-RBD-Wu-1), and anti-Omicron BA.1 RBD (anti-RBD-Omicron). (C) Pre-vaccination (T0) pseudotyped virus neutralization titers of BA.1, BA.2, BA.4, and XBB relative to Wu-1 stratified by previous Omicron exposure status (*n* = 16 without previous exposure, left; *n* = 29 with previous exposure, right). Fold changes are represented above the horizontal comparative lines. GMT of neutralizing antibodies is shown above each pseudotyped virus. Data points were compared using Friedman’s test with Holm-corrected post hoc Wilcoxon test. (D) Magnitude breadth analysis for neutralizing antibody responses pre-vaccination (T0) stratified by exposure to Omicron. Left: IgG anti-RBD-Omicron at T0 (dashed line represents anti-RBD-Omicron negative, solid line represents anti-RBD-Omicron positive). Right: area under the curve (AUC) for the individual magnitude-breadth curves. The error bars represent the mean and 95% confidence interval. Test significance determined by Wilcoxon rank-sum test. ∗*p* < 0.05; ∗∗*p* < 0.01; ∗∗∗*p* < 0.001; ∗∗∗∗*p* < 0.0001; absence of any (∗) = not significant. A minimum of *n* = 2 technical replicates were performed.

At study entry in January 2023, our population enrolled 101, prior unvaccinated, HIV negative participants in Abuja, Nigeria (cohort 1—primary cohort; Figure 1A). The median age was 33 years (inter-quartile range: 26–41) and the cohort was 51% female (Table 1). To assess prior exposure to SARS-CoV-2 at a time when Omicron was the dominant circulating variant, we measured IgG responses against ancestral Wu-1 antigens using a validated Luminex bead-based flow cytometric assay.^50^ Almost all participants were seropositive for pre-Omicron antigens: 94/101 (93%) for nucleocapsid (N), 98/101 (97%) for spike (S), and 97/101 (96%) for receptor-binding domain (RBD) (Figure 1B). This near-universal reactivity indicates widespread exposure to SARS-CoV-2 prior to vaccination rollout.

**Table 1:** Baseline characteristics of study participants in cohort 1

**Characteristic**	

Total number, *n* (%)	101 (100)
Age, median years (IQR)	33 (26, 42)
Female sex, *n* (%)	51 (51)
Time between vaccine doses, days (IQR)	62 (59, 65)

**Participants with neutralization response data**	

Total number, *n* (%)	45 (100)
Age, median years (IQR)	36 (28, 42)
Female sex, *n* (%)	25 (56)
Time between vaccine doses, days (IQR)	62 (59, 63)

To evaluate exposure to Omicron lineages, we next tested all participants for Omicron BA.1-specific IgG anti-RBD antibodies. Fifty-nine of 101 participants (58%) were positive (Figure 1B), suggesting that while nearly all had encountered pre-Omicron viruses, only a subset had been infected during the Omicron era. Based on genomic surveillance and reported cases in Nigeria (Figures S1A and S1B), these exposures most likely involved BA.1, BA.2, or BA.4 lineages, though infection with later subvariants such as BQ.1 or XBB.1.5 cannot be excluded. It is notable that BA.4 has two additional mutations over BA.2 at positions 452 and 486.^51^ This exposure pattern observed above aligns with national and regional epidemiological data. Since 2020, Nigeria’s SARS-CoV-2 landscape has been dominated sequentially by the ancestral strain (∼40%), Alpha/Eta (∼24%), Delta (∼18%), and Omicron subvariants (∼18%) from 2022 onward. Regionally, lineage dynamics were marked by Eta predominance over Alpha and the emergence of a rare Delta AY.36 sublineage.^52^ These data indicate that most study participants were likely primed by pre-Omicron variants before Omicron exposure, consistent with the high prevalence of antibodies to Wu-1 spike at study entry (Figure 1B).

### Validation of assay using an independent UK vaccine cohort

To rule out the possibility of residual Wu-1 cross-reactivity as a confounder in the Omicron exposure discriminatory capacity of our assay, we analyzed binding and neutralizing responses in an independent UK vaccine cohort (*n* = 21) who received four doses of Wu-1-based vaccine and were anti-N-negative prior to dose 3. Between doses 3 and 4—when Omicron lineage viruses were the predominant circulating strains in the United Kingdom (Figure S2A)—(*n* = 11) individuals experienced Omicron breakthrough infection (BTI), while (*n* = 10) remained infection-naïve (Figure S2B). We observed that BTI participants mounted a disproportionately stronger response to BA.1 neutralization (32.5-fold vs. 4.9-fold) and RBD-Omicron binding (2.6-fold vs. 1.4-fold) but a lower boost in Wu-1 neutralization compared with infection-naïve vaccinees (4.5-fold vs. 7.1-fold) (Figures S2C and S2D). The clear distinction in neutralization profiles between groups in Figure 1D, and the divergent patterns of responses in the UK control cohort supports the robustness of our screening assay and the utility of anti-RBD-Omicron as a reliable marker in identifying prior Omicron lineage exposure with minimal interference from Wu-1 cross-reactive antibodies.

### Pre-vaccination neutralization profiles indicate imprinted immunity

We first evaluated baseline serum neutralization responses to evaluate pre-vaccination imprinting. We used pseudotyped viruses as previously described^50^^,^^53^^,^^54^ in (*n* = 45) participants against ancestral Wu-1, BA.1, BA.2, BA.4, and the antigenically shifted XBB. XBB had not yet circulated in Nigeria at the time of sampling; minimal neutralizing activity against this variant was therefore expected. Characteristics and binding antibody data of this sub-population were comparable to the general cohort (Table 1; Figure 1B). Among participants with pre-Omicron exposure but no serological evidence of Omicron infection (anti-RBD-Omicron negative, *n* = 16), geometric mean neutralization titers (GMTs) were highest for Wu-1 (GMT = 1,243) and similar or lower for Omicron lineages (BA.1 = 658; BA.2 = 1,099; BA.4 = 1,387). Thus, BA.1 and BA.2 were 1.9-fold and 1.1-fold lower than Wu-1, while BA.4 was 1.1-fold higher relative to Wu-1. Furthermore, participants with evidence of prior Omicron exposure (anti-RBD-Omicron positive, *n* = 29) also exhibited higher neutralization titers to Wu-1 (GMT = 4281) than to Omicron BA.1 (GMT = 2,322), BA.2 (GMT = 2,104), and BA.4 (GMT = 2,112), corresponding to approximately 2-fold differences (Figure 1C).

Individuals recently infected with Omicron would be expected to exhibit the reverse pattern—i.e., higher titers against Omicron than Wu-1 given Omicron infection would be more recent. Instead, persistence of elevated Wu-1 titers suggests preferential recall of ancestral-strain antibodies. Neutralization against XBB was negligible across groups (GMT <100), confirming that activity was directed toward early Omicron lineages and provides additional supporting evidence of specificity of the screening IgG anti-RBD-Omicron antibody assay for early (pre-XBB) Omicron variants. There were a few Omicron-seronegative participants who demonstrated modest neutralization against Omicron subvariants, potentially reflecting exposure to either the Eta or Beta variants bearing E484 substitutions (as does Omicron), which confer escape to class I antibodies.

To confirm the discriminatory power of the anti-RBD-Omicron marker, we assessed magnitude and breadth of neutralizing antibody responses at baseline prior to vaccination. To quantify neutralization breadth, we generated magnitude-breadth curves using a Kaplan-Meier approach (Figure 1D). Compared with Omicron-exposed individuals, Omicron-naïve participants exhibited 2.9-fold (*p* < 0.05) lower magnitude-breadth scores. These data collectively indicate that antibody responses in the primary cohort were preferentially directed toward ancestral epitopes despite documented Omicron exposure.

### Confirmation of antibody recall to ancestral Wu-1 in a secondary Nigerian cohort

To confirm the observed phenomenon, we further characterized neutralization profiles in a second unvaccinated Nigerian cohort recruited in early 2023 as part of a SARS-CoV-2 fractional dosing clinical trial (PACT202206754734018)^54^^,^^55^^,^^56^ (cohort 2, Table S1). In this second cohort, a total of 64 samples were analyzed, of which, 94% (*n* = 60/64) showed serological evidence of Omicron exposure, defined by IgG anti-RBD-Omicron positivity (Figure S3A). This reflects a higher degree of exposure to Omicron relative to the primary cohort. Despite this, geometric mean neutralization titers (GMTs) against Wu-1 (GMT: 1,492) remained similar to or higher than against BA.1 Omicron (GMT: 843, 1.8-fold difference) and BA.2 (GMT: 1,316, 1.2-fold difference) (Figure S3B). The modestly higher BA.2 titers (∼1.6-fold relative to BA.1) likely reflect more recent exposure to BA.2 lineage viruses in 2023 (Figure S1B). Notably, Wu-1 neutralization titers remained robust despite more than 18 months elapsing since ancestral strain circulation, highlighting the persistence Wu-1 directed humoral responses. Taken together, these data from both Nigerian cohorts sampled under different epidemiological contexts support the presence of durable imprinted antibody patterns established by early pre-Omicron exposures.

### Depletion of serum antibodies against ancestral Wu-1 spike suggests imprinting

**Figure 2:**
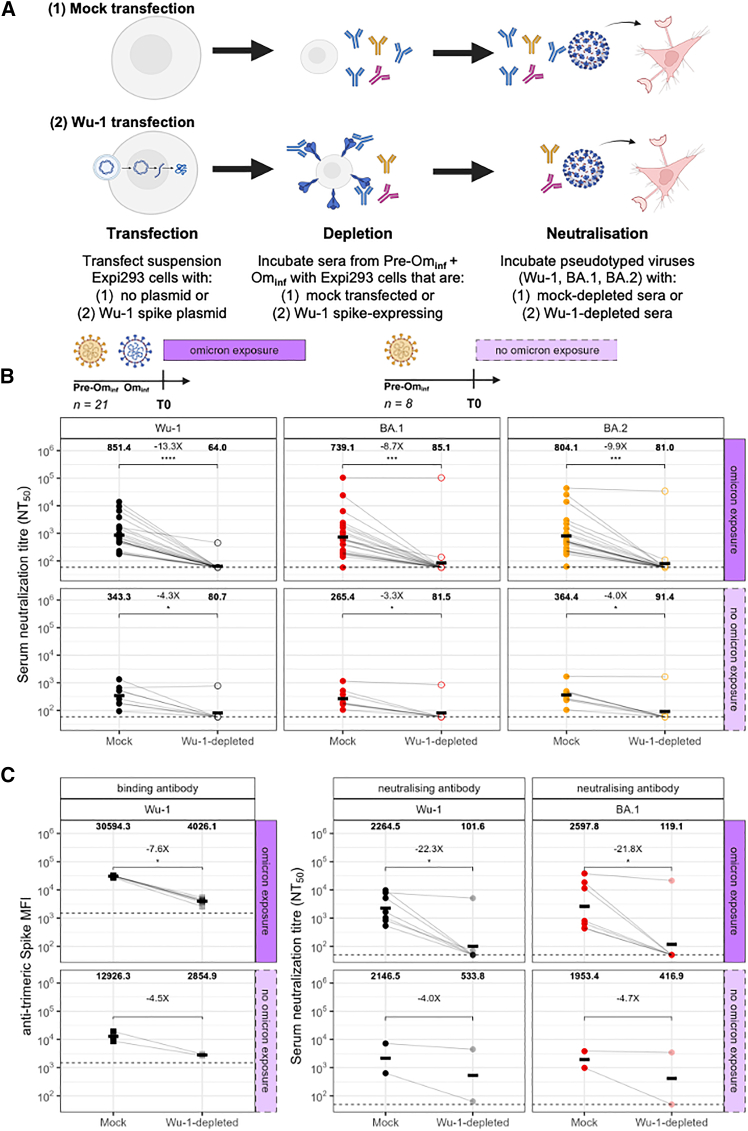
Demonstration of imprinted immunity using serum antibody depletion assay (A) Schematic of the experimental approach for Wu-1 spike depletion. Plasma samples were incubated with Expi293 cells expressing Wu-1 spike or a mock, non-transfected control, followed by neutralization and binding antibody assays to assess depletion efficiency. Created in BioRender. Morse, R (2026) https://BioRender.com/sc68hgs. (B) Pseudotyped virus neutralization titers against Wu-1, BA.1, and BA.2 at baseline (T0) in participants who were pre-Omicron exposed (*n* = 8) or both pre- and post-Omicron exposed (*n* = 21), comparing mock-depleted and Wu-1 spike-depleted sera. (C) In a subset (*n* = 9/29) of participants, including the (*n* = 2) individuals maintaining neutralization post-depletion, measurement of (left) binding antibody levels against Wu-1 spike using a Luminex assay (1/100 dilution) and (right) neutralization responses pre- and post-depletion. Each line represents an individual participant, with (*n* = 2) pre-Omicron-exposed participants and (*n* = 7) pre- and post-Omicron-exposed participants. Data are representative of two independent experiments comprised of two technical replicates. GMTs of neutralizing antibodies are shown above each depletion condition. Data points were compared using the Wilcoxon matched-pairs signed rank test with Holm’s multiple testing adjustment. ∗*p* < 0.05; ∗∗*p* < 0.01; ∗∗∗*p* < 0.001; ∗∗∗∗*p* < 0.0001; ns, not significant. A minimum of *n* = 2 technical replicates were performed.

To test the hypothesis that observed neutralization responses were driven by preferential recall of ancestral antibodies, we performed targeted depletion of plasma Wu-1 spike-specific antibodies from pre-vaccination sera. Samples were obtained from participants with exposure to pre-Omicron variants only (*n* = 8) and those with both pre- and post-Omicron exposures (*n* = 21) in the primary cohort. Using a previously described method,^34^ Wu-1 spike-binding antibodies were depleted using spike-expressing Expi293 cells (STAR Methods) and incubated for 72 h, after which participant sera were passed over the cells (Figure 2A). This process allowed surface-expressed spike to selectively bind and deplete Wu-1-specific antibodies from the serum. Residual neutralization activity was then assessed against Wu-1, BA.1, and BA.2 viruses.

Consistent with the hypothesis of infection-driven Wu-1 imprinting, depletion of Wu-1-specific antibodies resulted in reduction of neutralizing activity below limit of detection of neutralizing activity against Wu-1, BA.1, and BA.2 viruses in 7/8 (88%) pre-Omicron exposed participants and 20/21 (95%) pre- and post-Omicron exposed participants following depletion (Figure 2B). Successful depletion was confirmed by measuring Wu-1 spike-specific binding antibodies with Luminex in a subset (*n* = 9) of pre- and post-depletion samples that included two individuals who showed minimal change in neutralization activity following depletion (Figure 2C). No significant difference was observed between ages of participants who showed neutralization antibody abrogation (*n* = 27) relative to those that did not (*n* = 2) with median age of 35 years (*p* = 0.97).

### Identification of the RBD as the target of imprinting

**Figure 3:**
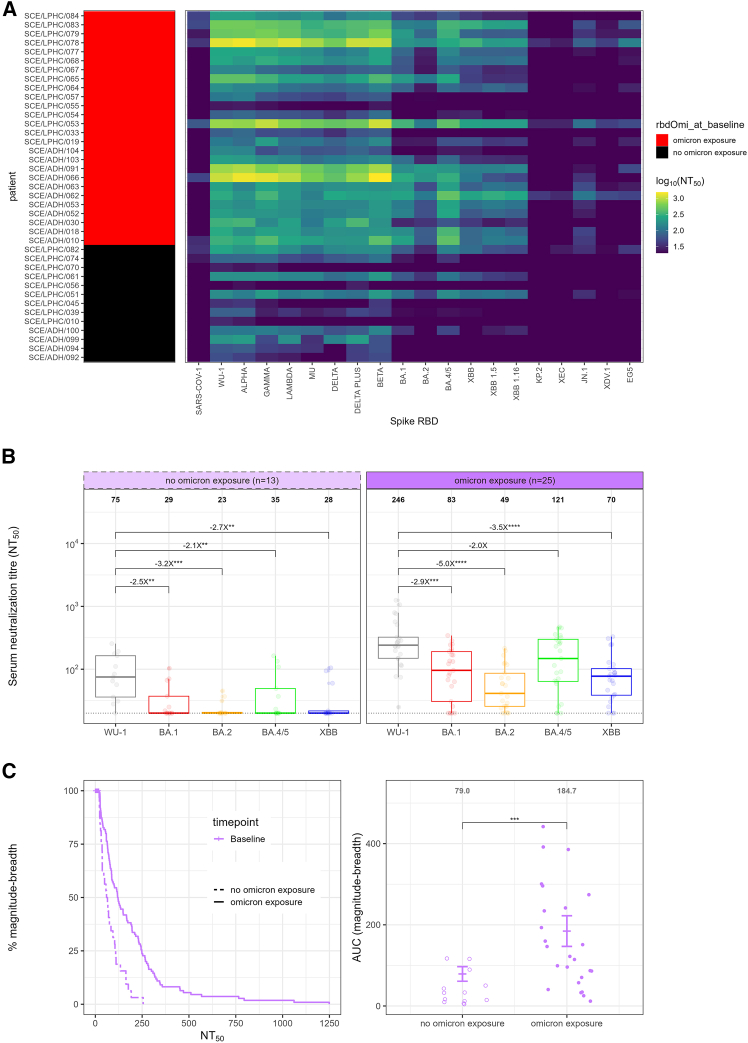
Surrogate serum neutralization assay using RBD from 20 VOCs demonstrates strong imprinting against the RBD (A) Pre-vaccination (T0) surrogate viral neutralization titer against the receptor binding domain (RBD) of SARS-CoV-1 and a series of SARS-CoV-2 variants of concern. The left heatmap represents the previous Omicron exposure status as determined by BA.1 RBD binding antibodies (red = with previous exposure, black = without previous exposure). The right heatmap represents the neutralization titer as log10(NT_50_) using a viridis color mapping. (B) Pre-vaccination (T0) surrogate viral neutralization titer against the receptor binding domain (RBD) of BA.1, BA.2, BA.4/5, and XBB relative to Wu-1 stratified by previous Omicron exposure status (*n* = 13 without previous exposure, left; *n* = 25 with previous exposure, right). Fold changes are represented above the horizontal comparative lines. GMT of neutralizing antibodies is shown above each pseudotyped virus. The boxplot represents the middle line represents the median, the hinges 25th and 75th percentiles, and whiskers are 1.5 x IQR from the hinge. Data points were compared using Friedman’s Test with Holm-corrected post hoc Wilcoxon test. (C) Magnitude breadth analysis for neutralizing antibody responses pre-vaccination (T0) stratified by exposure to Omicron. Left: IgG anti-RBD-Omicron at T0 (dashed line represents anti-RBD-Omicron negative, solid line represents anti-RBD-Omicron positive). Right: AUC for the individual magnitude-breadth curves. The error bars represent the mean and 95% confidence interval. Test significance determined by Wilcoxon rank-sum test. ∗*p* < 0.05; ∗∗*p* < 0.01; ∗∗∗*p* < 0.001; ∗∗∗∗*p* < 0.0001; absence of any (∗), not significant; ∗∗∗*p* < 0.001; ∗∗∗∗*p* < 0.0001; absence of any (∗), not significant.

To define the region of the spike protein primarily involved in infection-related imprinting, we tested sera from our unvaccinated cohort against RBD from 20 SARS-CoV-2 variants of concern using a surrogate neutralization assay. Serial dilutions of sera were incubated with purified RBD to measure inhibition of ACE2 in a Luminex bead environment (Figure 3A). Individuals previously identified as Omicron-exposed by the binding assay demonstrated greater breadth of neutralization against Omicron lineages compared to Omicron unexposed individuals. When geometric mean titers (GMTs) for Wu-1, BA.1, BA.2, and BA.4/5 were compared, titers against Omicron lineages were consistently lower than those against Wu-1 in both groups, demonstrating imprinting directed against the RBD (Figure 3B). Magnitude-breadth analysis further demonstrated that Omicron-exposed individuals (anti-RBD Omicron positive) had broader neutralization breadth against BA.1 and BA2 lineage spike RBD (Figure 3C), providing orthogonal validation of the binding antibody screening approach. Finally, antigenic maps of all 20 variants in the experiment revealed clustering of sera from both Omicron exposed and unexposed individuals around pre-Omicron lineages (Figure S4), consistent with imprinting toward ancestral RBD epitopes.

### Impact of Omicron breakthrough infection and contemporaneous Wu-1 vaccination

**Figure 4:**
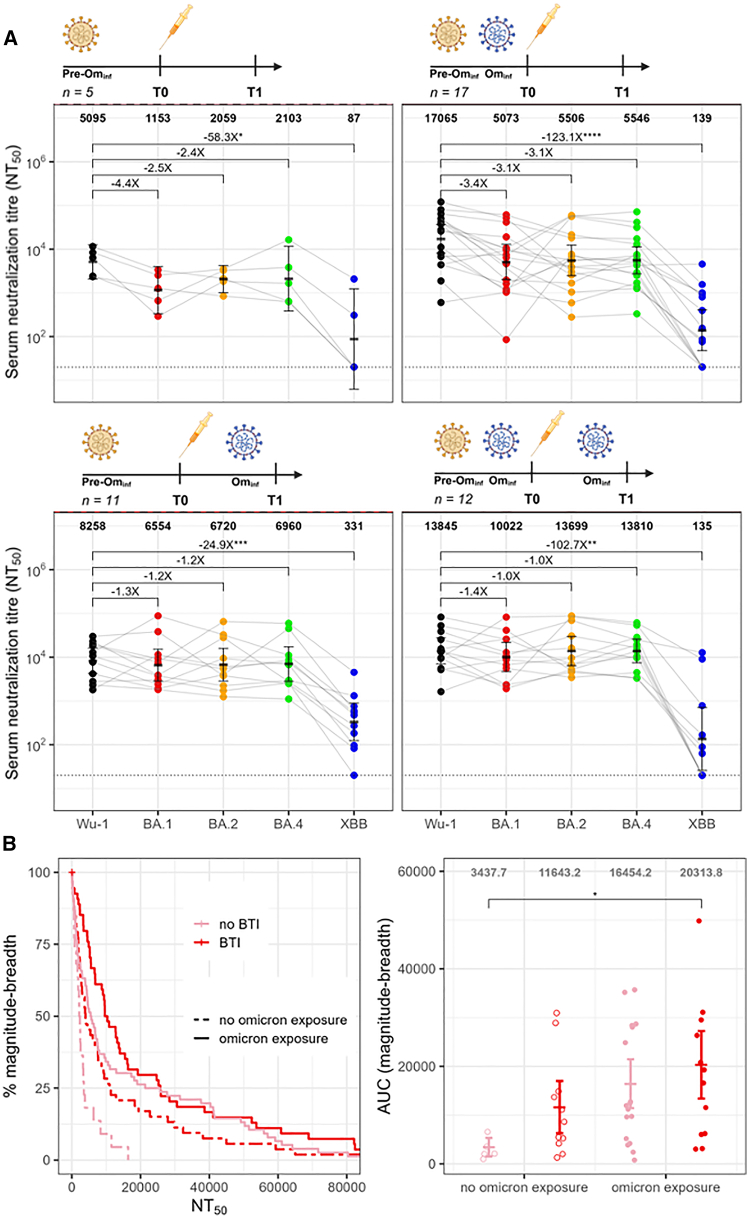
Neutralizing antibody responses to COVID-19 vaccination with or without breakthrough Omicron infection (A) Pseudotyped virus neutralization titers for BA.1, BA.2, BA.4, and XBB relative to Wu-1 at T1 (1-month post-dose 1) in participants without pre-vaccination Omicron exposure (left, *n* = 16) and with pre-vaccination Omicron exposure (right, *n* = 29). Further stratification by breakthrough infection (BTI) was performed at T1 (top, no BTI; bottom, BTI). GMTs of neutralizing antibodies are shown for each pseudotyped virus and fold changes relative to Wu-1 are shown above the horizontal comparative lines. Error bars represent the GMT and 95% confidence interval. Data points were compared using Friedman’s test with Holm-corrected post hoc Wilcoxon test. (B) Magnitude breadth curves comparing neutralization responses at T0 (pre-vaccination; dashed line, no Omicron exposure; solid line, Omicron exposure) and T1 (1-month post-dose 1; pink, no BTI; red, BTI). The *x* axis represents NT_50_ values, while the *y* axis represents the cumulative proportion of participants with neutralization above each threshold. Right: AUC for the individual magnitude-breadth curves. The error bars represent the mean and 95% confidence interval. Test significance determined by Kruskal-Wallis test with post hoc Dunn’s test. ∗*p* < 0.05; ∗∗*p* < 0.01; ∗∗∗*p* < 0.001; ∗∗∗∗*p* < 0.0001; absence of any (∗), not significant. A minimum of *n* = 2 technical replicates were performed.

In the primary cohort, *n* = 23 of 45 analyzed participants experienced a breakthrough infection between study entry and one month after the first dose (BTI; Figure S5). Among participants with prior Omicron exposure at baseline (*n* = 29), vaccination increased neutralization titers across ancestral and Omicron lineage regardless of BTI status (Figure S6A). In contrast, among those without prior Omicron exposure at baseline (*n* = 16), only participants who experienced BTI demonstrated a marked increase in neutralization titers across ancestral and Omicron lineage viruses, whereas responses in those without BTI rose modestly and were not significant (Figure S6B). These data suggest that Wu-1-based vaccination enhanced neutralizing activity against ancestral viruses, while Omicron exposure, whether prior or following vaccination, boosted responses to Omicron lineages. In participants without BTI, (*n* = 22), we observed that Wu-1-based vaccination further amplified ancestral bias. In Omicron-naïve individuals (*n* = 5), neutralization titers against Wu-1 (GMT: 5,095) were substantially higher than against Omicron variants BA.1 (1,153; 4.4-fold lower), BA.2 (2,059; 2.4-fold lower), BA.4 (2,103; 2.4-fold lower), and XBB (87; ∼58-fold lower) (Figure 4A [top, left]). A similar pattern was observed in participants with prior Omicron exposure (*n* = 17), where Wu-1 titers (GMT: 17,065) were roughly 3-fold higher than BA.1 (5,073), BA.2 (5,506), and BA.4 (5,546), and ∼123-fold higher than XBB (139).

We next investigated individuals who experienced BTI after vaccination (*n* = 23/45). Omicron was the dominant circulating variant (Figure S1B) and was likely responsible for the BTI. In participants with no prior Omicron exposure at baseline (*n* = 11), following contemporaneous Wu-1-based vaccination and Omicron BTI, titers were comparable across Wu-1 (GMT: 8,258), BA.1 (GMT: 6,554; 1.3-fold lower), BA.2 (GMT: 6,720; 1.2-fold lower), and BA.4 (GMT: 6,960; 1.2-fold lower), with XBB remaining poorly neutralized (GMT: 331; ∼25-fold lower) (Figure 4A [bottom, left]). In individuals with both prior Omicron exposure and BTI (*n* = 12), titers were also broadly equivalent relative to Wu-1 (GMT: 13,845), BA.2 (GMT: 13,699), and BA.4 (GMT: 13,810), with BA.1 modestly lower (GMT: 10,022; 1.4-fold difference) and XBB markedly reduced (135; ∼103-fold lower) (Figure 4A [bottom, right]). The slightly lower BA.1 titers are consistent with BTIs caused by BA.2-descendant lineages.

To quantify how Wu-1 vaccination and Omicron exposure shaped antibody breadth, we generated magnitude-breadth curves of responses. Magnitude-breadth analysis showed a 3-fold increase at one-month post-dose 1 (T1) in Omicron-naïve and a 2-fold increase in Omicron-exposed participants relative to baseline (T0) (Figure 4B) indicating enhanced neutralization capacity across both groups, with a greater relative boost in the Omicron-naïve group. Stratification by BTI status showed that participants with no Omicron exposure at any time point (*n* = 5) had persistently lower magnitude-breadth than those with at least one Omicron exposure (5.9-fold difference, *p* = 0.03). The sequence of Omicron exposure influenced outcomes: pre-vaccination Omicron exposure without BTI (*n* = 17) exhibited greater magnitude-breadth than post-vaccination Omicron BTI alone (*n* = 11, *p* < 0.05), while combined pre- and post-vaccination exposure mounted the broadest and most balanced responses (Figure 4B). These findings suggest that Wu-1-based vaccination alone reinforced ancestral-biased immunity, whereas hybrid (infection and vaccine)-induced immunity with repeated Omicron exposure—either before or after vaccination partially mitigate of imprinting and broadened the antibody response.

## Discussion

Understanding how sequential SARS-CoV-2 exposures shape immune responses is critical for anticipating population-level protection and informing future vaccine strategies. Immune imprinting, where primary exposure to an ancestral variant constrains subsequent responses to antigenically distinct strains is now recognized as a major determinant of neutralization breadth.^54^^,^^57^^,^^58^^,^^59^^,^^60^^,^^61^^,^^62^^,^^63^^,^^64^ The implications are substantial: as pathogens evolve greater immune escape and pathogenicity, prior imprinting may limit the induction of effective *de novo* neutralizing responses. Most available human data reflect vaccine-first exposure histories, since global vaccination campaigns preceded the emergence of the Omicron variant. By contrast, delayed vaccine rollout across Africa created a natural experiment where repeated infection-driven exposures occurred before first vaccination. We leveraged this to investigate the possibility of infection-induced imprinting and mitigation by subsequent Omicron exposure in two unvaccinated Nigerian cohorts.

Our data are consistent with infection-induced immune imprinting in the absence of prior vaccination. Nearly all participants from the primary cohort demonstrated evidence of pre-Omicron infection and neutralization response profiles confirmed dominant recall of ancestral Wu-1 responses even among those subsequently infected with Omicron. These data are consistent with our previous work and other studies from Nigeria and Ghana showing high seroprevalence for Wu-1 binding antibodies and robust serum neutralizing activity in 2021, alongside high rates of BTI by the Delta variant among vaccinated healthcare workers.^19^^,^^50^^,^^65^^,^^66^ In Ghana, a large cross-sectional study conducted in late 2023 found 99% spike seropositivity and plasma neutralization favoring Wu-1 over Omicron variants, despite widespread exposure^67^—further evidence of immunological imprinting by ancestral SARS-CoV-2 in a West African population. In our study, participants with serological evidence of Omicron exposure paradoxically showed similar or lower neutralization of Omicron compared with Wu-1, despite Omicron being the more recent exposure.

A second contemporaneous unvaccinated Nigerian cohort reinforced this observation: despite 94% Omicron exposure, neutralization titers showed the same patterns with Wu-1 neutralizing titers exceeding Omicron titers—suggesting durable recall of ancestral memory B cells instead of *de novo* Omicron responses. Depletion experiments directly confirmed that Wu-1-binding antibodies were responsible for abrogation of neutralization against both Wu-1 and Omicron-lineages in 27/29 of tested participants. Repeated Omicron infection after Wu-1 vaccination was observed to partially equalize titers between Wu-1 and Omicron but did not allow Omicron responses to surpass ancestral ones, indicating only partial mitigation of imprinting.

These findings parallel findings from animal models and Wu-1-based vaccine studies, showing that both vaccination and infection can drive strong recall of conserved epitopes while limiting generation of Omicron-specific responses.^27^^,^^68^^,^^69^ Globally, individuals primed with Wu-1-based vaccines and who later experienced Omicron infections predominantly recalled pre-existing memory B cells recognizing conserved epitopes rather than generating *de novo* Omicron-specific clones, as evidenced by comparable neutralizing titers against ancestral and Omicron strains.^25^^,^^26^^,^^27^ BCR-repertoire studies support this recall-dominant pattern,^70^^,^^71^ with breakthrough infections in individuals primed with ancestral vaccines likely to show preferential recall of conserved, high-affinity class-switched memory B cell clones with extensive somatic hypermutation, while recruitment of naive B cells specific for Omicron-unique RBD epitopes remains limited. In one study, individuals primed with the inactivated CoronaVac vaccine and subsequently infected twice with Omicron developed comparable neutralizing titers against ancestral and Omicron strains and expanded Omicron-specific memory B cells, suggesting partial mitigation of imprinting.^69^ In contrast, individuals who received at least three doses of Wu-1 mRNA vaccine and later experienced Omicron BTI or XBB.1.5 monovalent vaccination maintained the highest titers against Wu-1 with minimal Omicron-specific memory B cell induction.^36^^,^^68^ Supporting our depletion data, absorption of Wu-1 binding antibodies in these studies abrogated BA.5 and XBB.1.5^36^^,^^68^ neutralization, mirroring our findings where depletion of Wu-1 antibodies abrogated neutralization against BA.1 and BA.2. This confirms that the Omicron neutralization response largely reflects cross-reactive recall and expansion of ancestral Wu-1-imprinted memory B cells rather than generation of *de novo* Omicron-specific responses.

Notably, two individuals in our study retained neutralizing activity against both Wu-1 and Omicron even after removal of Wu-1 spike-binding antibodies. Previous work has demonstrated *de novo* antibody generation after multiple Omicron exposures in individuals who received inactivated or recombinant SARS-CoV-2 vaccination.^37^^,^^69^ On this basis, one explanation for our observation could be the emergence of new antibody clones targeting subdominant or cryptic epitopes of spike that are not efficiently depleted by Wu-1 spike binding. Alternatively, these antibodies may recognize conformational epitopes exposed only after ACE2 engagement or spike trimer rearrangement,^72^ allowing residual neutralization despite Wu-1 antibody removal.

Regarding mitigation of imprinting, participants, exposed to Omicron both before and after Wu-1-based vaccination showed near-equal neutralization titers for Wu-1 and BA.1/BA.2/BA.4 variants. In contrast, exposed participants without post-vaccine BTI retained a 3-fold ancestral bias, i.e., 3-fold lower titers against Omicron variants. These data indicate that infection-induced imprinting can be partially mitigated but not eliminated by repeated antigenic challenge with divergent variants.

In summary, our study provides one of the first detailed demonstrations of infection-induced immune imprinting to SARS-CoV-2. We show that infection in the absence of vaccination can induce durable immune imprinting comparable to that caused by Wu-1-based vaccination with repeated Omicron exposure only partially broaden antibody breadth without fully overcoming the ancestral bias. These findings highlight the importance of incorporating data from underrepresented regions into global immunological understanding and support the deployment of variant-adapted vaccines to override imprinting by ancestral pre-Omicron strains and provide broader protection for vulnerable populations such as the elderly or those with compromised immunity.

Beyond SARS-CoV-2, these insights are relevant to other rapidly evolving viral families, including other sarbecoviruses and influenza viruses, where sequential exposures shape immune memory and influence vaccine efficacy.^60^^,^^62^^,^^73^^,^^74^ Finally, as the risk of long COVID, post-infection sequelae and complex infection-vaccine exposure sequences arise with each infection^75^^,^^76^ recognizing and mitigating imprinting will be central to sustaining effective protection against evolving SARS-CoV-2 variants. Future work should assess how host factors, including HIV infection^77^^,^^78^^,^^79^ and other causes of immune dysregulation, alters imprinting, antibody breadth, and responses to sequential infection-vaccination exposures.

### Limitations of the study

Our study was subject to limitations. We lacked direct genomic confirmation of infecting variants and did not assess T cell responses. However, contemporaneous epidemiological and serological data from Nigeria strongly support our inferred exposure histories of Wu-1 followed by Omicron infection. Given that up to half of SARS-CoV-2 infections are asymptomatic,^75^^,^^76^ it is impractical to rely solely on clinical case confirmation as recent data also indicates for re-emerging infections such as mpox virus.^80^ Instead, we validated the use of serology, specifically binding and neutralizing antibody profiling, to determine pre- and post-Omicron variant exposure that reflected the limited population-level genomic surveillance data available in Nigeria. We additionally note that although there are still no universally accepted clinical correlates of protection for Omicron, we observed relatively high absolute neutralizing antibody responses against multiple Omicron sub-lineages using our pseudotype virus assay. One explanation for this is that absolute neutralization titers measured by pseudovirus assays can run up to ∼2 log_10_ higher than live-virus values^81^^,^^82^ and may not be directly comparable with live-virus assays^83^^,^^84^^,^^85^^,^^86^ and therefore, our interpretations rely on within-assay fold changes, where neutralization of Wu-1 remained consistently ∼2-fold higher than BA.1/BA.2/BA.4 despite recent Omicron exposure. While such imprinting deficits are unlikely to have immediate clinical impact in young immunocompetent adults such as our cohort with a median age of 35 years where peak titers are well above putative protection threshold but may reduce the “protection-margin” as titers wane or in older or immunocompromised populations. Finally, our RBD discriminatory assay was based on BA.1, which contains all nine conserved mutations shared across later Omicron sublineages ensuring broad antigenic coverage and robust discrimination of exposure history over pre-Omicron exposure.^25^^,^^27^^,^^87^^,^^88^^,^^89^ This therefore represents a conservative approach that provides a lower-bound estimate of Omicron but remains robust in discriminating exposure.

## Resource availability

### Lead contact

Further information and requests for resources and reagents should be directed to and will be fulfilled by the lead contact, Ravindra K. Gupta (rkg20@cam.ac.uk).

### Materials availability

This study did not generate new unique reagents.

### Data and code availability

• Data reported in this paper are available from the lead contact upon reasonable request.
• This paper does not report original code.
• Any additional information required to reproduce or further analyze the results presented in this paper are available from the lead contact upon reasonable request.

## Acknowledgments

The authors wish to thank the volunteers for their participation in this study and the staff at Lugbe Primary Health Center, Asokoro District Hospital, and Maitama District Hospital in Abuja Nigeria. A.A. was supported by the Cambridge-Africa Alborada award and Harvard Takemi Program in International Health. R.B.M. was supported by the Harding Distinguished Postgraduate Scholars Programme. D.J.S. and S.T. are supported by the NIH NIAID Centers of Excellence for Influenza Research and Response (CEIRR) contract 75N93021C00014 as part of the SAVE program. D.J.S. and S.T. are supported by the NIH NIAID Centers of Excellence for Influenza Research and Response (CEIRR) contract 75N93021C00014 as part of the SAVE program. R.K.G. was supported by a 10.13039/100010269Wellcome Trust Senior fellowship (WT108082AIA). This research was supported by the Hong Kong Jockey Club Global Health Institute (HKJCGHI), Hong Kong Special Administrative Region, China. This research was supported by the 10.13039/501100018956NIHR Cambridge Biomedical Research Centre (NIHR203312) and Open Philanthropy.

## Author contributions

Study conceptualization and design, A.A. and R.K.G.; study management, supervision, and fieldwork, A. Abdullahi., E.O., M.E., and A. Abimiku.; methodology and investigation, A.A., R.B.M., E.O., M.T.K.C., M.E., S.T., F.I., A.K., B.S., H.W.A., E.J., O.B., A.S., H.A.-O., A.J., A.M., O.O., B.I., O.E., A. Anzaku., E.O., S.O., I.M.K., S.H.A., B.M., L.R., R.D., W.W., D.S., and B.S.; research data curation and analysis, A.A., R.B.M., M.T.K.C., F.I., A.K., B.S., A.Ab., and R.K.G.; funding acquisition, A.A., A.Ab., B.S., and R.K.G.; writing, A.A. drafted the original manuscript and R.B.M. provided critical editorial input, with contributions from all authors. All authors approved the final version of the manuscript.

## Declaration of interests

The authors declare no competing interests.

## STAR★Methods

### Key resources table

**Table:** 

REAGENT or RESOURCE	SOURCE	IDENTIFIER
**Bacterial and virus strains**		

XL10-Gold Ultracompetent Cells	Agilent	Cat#200314

**Biological samples**		

Human sera	as described in study population and sampling	

**Chemicals, peptides, and recombinant proteins**		

Recombinant SARS-CoV-2 spike RBD proteins from Wu-1 D614G	Sino Biological	#40592-V08H
Recombinant SARS-CoV-2 spike RBD proteins from Omicron (B.1.1.529)	Sino Biological	#40592-V08H121
Recombinant SARS-CoV-2 trimeric spike protein (S) from Wu-1 D614G	Abdullahi et al.^19^	
Recombinant SARS-CoV-2 nucleocapsid protein (N) from Wu-1 D614G	Abdullahi et al.^19^	
AviTag-biotinylated SARS-CoV-2 RBD-conjugated MagPlex-Avidin beads (multiple variants)	Tan et al.^96^	
PE-conjugated human ACE2	Tan et al.^96^	

**Critical commercial assays**		

Bright-Glo	Promega	Cat#E2650
Bio-Plex 200 system	BioRad	Cat#171000201

**Experimental models: Cell lines**		

HEK293T	ATCC	Cat#CRL-3216
HeLa-ACE2	James Voss	
Expi293F	ThermoFisher Scientific	Cat#A14527

**Software and algorithms**		

R v4.4.1	Rstudio	https://www.r-project.org/
Racmacs v1.2.8	Sam Wilks	https://acorg.github.io/Racmacs/
mafft v7.525	Open source (conda)	
Nextstrain	Hadfield et al.^98^	
outbreak.info R Client	Manar Alkuzweny and Karthik Gangavarapu and Laura Hughes	https://outbreak-info.github.io/R-outbreak-info
Survival v.3.8.3	Terry Therneau	https://CRAN.R-project.org/package=survival
Survminer 0.5.0	Alboukadel Kassambara and Marcin Kosinski and Przemyslaw Biecek	https://rpkgs.datanovia.com/survminer/index.html
AdjustedCurves v0.11.2	Denz et al.^108^	
PRISM v9.3.1	Graphpad	
Rstatix v0.7.2	Alboukadel Kassambara	https://rpkgs.datanovia.com/rstatix/

### Experimental model and study participant details

#### Human subject study population and sample collection

##### Cohort 1

101 HIV Negative participants across three clinical sites affiliated with Institute of Human Virology Nigeria (IHVN), Abuja, were actively recruited and asked to participate in this study. Eligible participants were men and non-pregnant women >18 years old who had no previous SARS-CoV-2 vaccination and were confirmed HIV negative using the Nigerian national HIV rapid testing algorithm.^90^^,^^91^ As interim recommendations and guidelines highlighted the use of a two-dose regimen of Ad26.COV2.S vaccine given two months apart due to improved vaccine efficacy,^92^ participants were administered a second dose two months following the first dose. Following signed informed consent, participants were recruited in this prospective observational cohort study. Participants were not randomized; eligible adults to receive their first-dose vaccination between 23^rd^ January 2023 to 20^th^ April 2023. Participants were recruited by i) local community outreach and ii) through phone calls to previously registered patients across three health facilities in Abuja, Nigeria. Participants provided plasma samples at baseline (prior to first-dose, T0) and 1-month post-dose 1 (T1). Baseline characteristics are shown in Table 1. Gender, ancestry, race, ethnicity, and socioeconomic status were not reported. Where applicable, participants were stratified post hoc based on serological evidence of SARS-CoV-2 exposure (e.g., Omicron-exposed vs non-exposed) for downstream analyses.

##### Cohort 2

Pre-vaccination samples from 64 individuals in the SIFCoVAN trial across Nigeria recruited in 2023 were tested for binding antibodies and pseudotyped virus neutralization across VOC. The protocol was registered with the Pan African Clinical Trials Registry (PACTR) PACTR 202206754734018. Participants were not randomized; samples were analyzed as an observational dataset. Allocation to analytical groups was performed retrospectively based on serological profiling (e.g., variant-specific binding and neutralization responses). Baseline characteristics are shown in Table 1. Gender, ancestry, race, ethnicity, and socioeconomic status were not reported.

#### Cell lines

HeLa cells stably expressing ACE2 (HeLa-ACE2) and human embryonic kidney 293T (HEK293T) cells were cultured in Dulbecco’s Modified Eagle’s Medium (DMEM, Gibco) supplemented with 10% fetal bovine serum (FBS) and 1% penicillin/streptomycin. Cells were maintained in an incubator at 37°C and 5% CO_2_. Expi293F suspension cells were cultured in Expi293 Expression medium (Gibco). Cells were maintained at 37°C and 8% CO2 with constant rocking in 125-ml vented Erlenmeyer flasks. Cell line identity was as provided by the source laboratories, and no additional authentication was performed in this study. All cell lines were routinely tested and confirmed to be free of mycoplasma contamination prior to use.

### Method details

#### Laboratory methods and sample testing

##### Binding antibody measurement

Binding IgG antibodies (Abs) against SARS-CoV-2 trimeric spike protein (S), nucleocapsid protein (N), Wu-1 D614G and Omicron (BA.1)-specific receptor-binding domain (RBD) were measured using the Luminex-based SARS-CoV-2-IgG assay by flow cytometry as previously detailed.^19^^,^^50^^,^^65^^,^^66^ The assay was validated using pre-pandemic serum samples and robustness of the defined cut-off is shown in Figure S7. We defined positive total anti-S antibody (anti-S) as anti-S IgG above cut-off of 226.48 mean fluorescence intensity (MFI), positive total RBD as anti-RBD-Wu-1 above cut-off of 411.9, and positive total anti-RBD-Omicron above cut-off of 729. Cut-offs were defined based on analysis of ‘true’ positive (convalescent) and negative pre-pandemic samples. We defined previous SARS-CoV-2 infection as positive anti-N IgG above a cut-off of 1472.8.

##### Neutralization assays

As previously described in detail,^53^^,^^93^ XL10-Gold ultracompetent cells (Agilent) were transformed with plasmids of interest (Wu-1 D614G, BA.1, BA.2, BA.4 and XBB) and DNA was harvested. To generate lentiviral SARS-CoV-2-pseudotyped viruses, HEK293T cells were transfected with plasmids for Wu-1 D614G, BA.1, BA.2, BA.4 and XBB. Pseudotyped viruses were titrated and diluted to ∼400,000 relative light units before incubation with human sera for 1 hour. The pseudotyped virus and sera mixture was then added to HeLa-ACE2 cells and incubated for 48 hours at 37°C and 5% CO_2_. All plates were read using Bright-Glo (Promega) and the Promega Glomax machine. There is evidence to show a high correlation between pseudotyped virus and live virus neutralization.^94^^,^^95^ All neutralization assays were repeated in two independent experiments containing two technical replicates for each condition.

#### Antibody depletion assay

##### Expi293F cell transfection

Expi293F cells were sub-cultured at least three times before transfection. Four days prior to experiments, cells were passaged to a density of 2.5-3 x 10^6^ viable Expi293F cells/ml. Three days prior to experiments, Expi293F cells were transfected with a mixture of Opti-MEM Reduced Serum Medium, Fugene HD transfection reagent, and either plasmids expressing Wuhan-Hu-1 spike or no plasmid (mock transfection control).

##### Serum absorption

One day prior to serum absorption-neutralization experiments, 96-well F-bottom cell culture microplates were plated with 2 x 10^4^ HeLa-ACE2 cells per well. On the day of the experiment, 72 hours post-transfection, Expi293F cells were centrifuged and washed twice with warm Expi293 Expression Medium, then resuspended at 40 x 10^6^ cells/mL. Heat-inactivated serum samples were initially diluted 1:10 in warm Expi293 Expression Medium and split into two fractions for absorption by Wuhan-Hu-1 spike-transfected Expi293F cells or mock-transfected Expi293F cells.

For each individual, Expi293 Expression Medium containing 8 x 10^6^ Expi293F cells were mixed 1:1 (v/v) with 1:10 diluted serum in 1.5-ml microcentrifuge tubes. The mixtures were incubated for 1 hour at 37°C and shaken at 900 rpm in an Eppendorf Thermomixer. After incubation, Expi293F cells were centrifuged, and the supernatant was transferred to a new microcentrifuge tube for re-centrifugation. Expi293 Expression Medium was added to the clarified supernatant such that all absorbed serum samples were now diluted 1:20.

Absorbed serum with a dilution of 1:20 were then added to 96-well cell culture F-bottom plates in duplicate and serially diluted 1:3 in DMEM supplemented with 10% FBS. Pseudotyped Wuhan-Hu-1, BA.1, and BA.2 viruses were added as previously described to the serial dilutions of absorbed sera, mixed and incubated at 37°C and 5% CO2 for 1 hour. After the incubation of absorbed sera and pseudotyped viruses, these mixtures were added to the plates of HeLa-ACE2 cells prepared the day prior to experiments. The final dilution of serum was 1:58.33. Neutralization plates were incubated and read as previously described.

#### Surrogate neutralization assay with panel of RBDs from VOC

We performed multiplex sVNTs with AviTag-biotinylated SARS-CoV-2 RBD-conjugated MagPlex-Avidin beads (Luminex) and PE-conjugated human ACE2 (a kind gift from Chee-Wah Tan) as previously described.^96^ In brief, heat-inactivated human serum samples at a starting dilution of 1:10 were titrated on a 96-well plate using a four-point, four-fold dilution series in assay buffer (1% BSA + 1 mM NaCl in 1X PBS). SARS-CoV-2 RBD-coated beads (600 beads per antigen) were mixed and incubated with the serum dilution series for a final dilution of 1:20 on the top row of the 96-well plate. Sera and beads were incubated for 1 hour at 25°C and 300 rpm. Next, 50 uL of 1000 ng/ml PE-conjugated ACE2 was added to each well and incubated for 1 hour at 25°C and 300 rpm. Finally, the plate was incubated on a magnetic plate holder for 2 minutes and washed twice with 1% BSA in 1X PBS before reading on the Bio-Plex 200 system (BioRad).

##### Antigenic cartography

The antigenic map was constructed using antigenic cartography with the R package Racmacs (v1.2.8) [https://doi.org/10.1126/science.1097211] in R (v4.4.1) using surrogate neutralization data. The table of neutralizing titers is converted into a distance table by calculating log_2_fold change from the maximum titer for each serum to all other titers per serum. The coordinate map for each serum and variant is then optimized in 2000 steps. The Racmacs::mapDistances function was used to extract antigenic distances of each serum sample from each variant.

##### RBD alignment

The Spike sequences for Wuhan-Hu-1 (NC_045512.2), BA.1 (OL672836.1), BA.2 (OM371884.1), BA.4 (ON373214.1), BA.2.86 (OR775659.1) and XBB (XBB 1.5 OP790748.1 + S: P486S) were aligned using MAFFT.^97^ SARS-CoV-2 spike RBD residues 319-541 were aligned. Asterisks indicate completely conserved columns in the alignment. The receptor binding motif (RBM) at residue 438-506 is indicated by + highlighted in yellow.

#### Epigenomic map

Genomic epidemiological data from GISAID was representatively sampled and visualized using Nextstrain.^98^ To further break down the cases in Nigeria in both the pre-Omicron and post-Omicron study periods, the Global initiative on Sharing All Influenza Data (GISAID) database^99^ was queried using Outbreak.info API^100^ for all SARS-CoV-2 sequences collected from Nigeria. Subclades of WHO-assigned Variants of Concern (VOCs) were assigned their WHO name and Nextstrain-assigned clade. To account for the changing number of SARS-CoV-2 cases, we weighed the number of sequences attributed to each VOC by weekly new cases reported to the WHO (https://data.who.int/dashboards/covid19/data):

$${Prevelance}_{\left(variant\right)}=\frac{{New\;Cases}_{\left(variant\right)}}{{New\;Cases}_{\left(total\right)}}$$

Where the weighted new cases attributed to a variant is derived by the weekly summation:

$${New\;Cases}_{\left(variant\right)}=\sum_{w}\left(\frac{{Sequences}_{\left(variant,w\right)}}{{Sequences}_{\left(total,w\right)}}\times {New\;cases}_{\left(w\right)}\right)$$

Where ***w*** is the week.

#### Magnitude breadth analysis

To capture the relationship between the magnitude (NT_50_) and breadth (percentage of VOCs neutralized) of serum neutralization, we plotted magnitude-breadth curves, a framework previously used in analyzing HIV^101^^,^^102^^,^^103^ and SARS-CoV-2^104^ humoral responses against multiple strains. Magnitude-breadth curves are Kaplan-Meier curves using the neutralization data against all tested variants, where the “event of interest” is the failure of neutralization of any variant, and the “time-to-event” on the x-axis is the NT_50_ for the same variant. Curves are modelled using the R package Survival v3.8-3,^105^^,^^106^ and plotted using the R package survminer 0.5.0.^107^ In addition, the areas under the curve (AUC) were calculated for both individual curves and aggregated curves were calculated using the “km” method in package AdjustedCurves v0.11.2 to facilitate comparison.^108^

### Quantification and statistical analysis

We defined vaccine breakthrough infection as a ≥2-fold increase in IgG anti-RBD-Omicron levels relative to study entry timepoint (Figure S5). Baseline characteristics of participants were expressed as proportions and percentages for categorical variables and median inter-quartile range (IQR) for continuous variables. 50% serum neutralizing titers (NT_50_) were plotted across timepoints with Geometric Mean Titer (GMT) indicated. For pseudovirus neutralization assays, NT_50_ were compared using Friedman’s Test with Holm-corrected post-Hoc Wilcoxon test. Neutralization data represented as boxplots (Figures 1C and 3B) included a middle line representing the median, hinges at the 25^th^ and 75^th^ percentiles, and whiskers at 1.5 x IQR from the hinge. Neutralization data in Figure 4A were plotted as the GMT and 95% confidence interval. For comparison of binding and neutralization in mock and Wu-1-depleted sera, we performed the Wilcoxon matched-pairs signed rank test with Holm’s multiple testing adjustment. Area under the curve was calculated for individual magnitude-breadth curves with error bars representing the mean and 95% confidence interval. For comparison of magnitude-breadth curves in individuals with and without Omicron exposure, test significance was determined by Wilcoxon rank-sum test (Figures 1D and 3C). For comparison of magnitude-breadth curves for individuals with and without Omicron exposure, as well as with and without breakthrough infection, test significance was determined by Kruskal-Wallis test with post-hoc Dunn’s test. P-values were reported with asterisks as follows: ∗*P* < 0.05; ∗∗*P* < 0.01; ∗∗∗*P* < 0.001; ∗∗∗∗*P* < 0.0001; absence of any (∗) = not significant. Statistical analysis was performed using GraphPad Prism version 9.3.1 and Rstatix v0.7.2.

### Additional resources

#### Ethics

This study was approved by the FCT Health Research Ethics Committee (FCT HREC) with approval FHREC/2022/01/193/18-10-22 and London-Surrey Research Ethics Committee [23/PR/0586] with IRAS ID: 309527. Approvals were also obtained from the secondary health centers – Asokoro District Hospital [Approval ID: FCTA/HHSS/HMB/ADH/111/22] and Maitama District Hospital [Approval ID: FCTA/HHSS/HMB/GEN/038/T].
