# Supplementary material for: SARS-CoV-2 Omicron infection reveals imprinted antibody responses in the absence of vaccination
Source: iScience. 2026 Apr 28;29(6):115910. doi: 10.1016/j.isci.2026.115910 (PMC13218260; doi:10.1016/j.isci.2026.115910)
Supplement: Document S1. Figures S1–S7 and Tables S1 and S2 [file mmc1.pdf]

iScience, Volume *29*

Supplemental information

SARS-CoV-2 Omicron infection reveals imprinted antibody responses in the absence of vaccination

Adam Abdullahi, Rebecca B. Morse, Mark Tsz Kin Cheng, Fehintola Ige, James Onyemata, Martin Edun, Anezka Kramna, Benjamin Sievers, Sam Turner, Haruna Wisso, Emmanuel Jonathan, Olasinbo Balogun, Abideen Salako, Hafsat Abdulazeez-Oha, Ayorinde James, Adesola Musa, Onisile Oluwaseun, Bamidele Iwalokun, Oliver Ezechi, Abbas Anzaku, Evaezi Okpokoro, Sophia Osawe, Gospel Nwikue, Ibrahim M. Kida, Baba Maiyaki Musa, Hassan Adam Murtala, Bo Meng, Leah Rosenzweig, Sani H. Aliyu, Derek Smith, Paul MacAry, Rainer Doffinger, Witold Więcek, Babatunde Salako, Chee Wah Tan, Alash'le Abimiku, and Ravindra K. Gupta

a

```
                    301......310.......320.......330.......340.......350.......360

Wuhan-hu-1   301    ------------------RVQPTESIVRFPNITNLCPFGEVFNATRFASVYAWNRKRISN
BA.1         301    ------------------RVQPTESIVRFPNITNLCPFDEVFNATRFASVYAWNRKRISN
BA.2         301    ------------------RVQPTESIVRFPNITNLCPFDEVFNATRFASVYAWNRKRISN
BA.4         301    ------------------RVQPTESIVRFPNITNLCPFDEVFNATRFASVYAWNRKRISN
XBB          301    ------------------RVQPTESIVRFPNITNLCPFHEVFNATTFASVYAWNRKRISN
BA.2.86      301    ------------------RVQPTESIVRFPNVTNLCPFHEVFNATRFASVYAWNRTRISN
consensus    301    ------------------*************I******d******r*********k****

                    361......370.......380.......390.......400.......410.......420

Wuhan-hu-1   361    CVADYSVLYNSASFSTFKCYGVSPTKLNDLCFTNVYADSFVIRGDEVRQIAPGQTGKIAD
BA.1         361    CVADYSVLYNLAPFFTFKCYGVSPTKLNDLCFTNVYADSFVIRGDEVRQIAPGQTGNIAD
BA.2         361    CVADYSVLYNFAPFFAFKCYGVSPTKLNDLCFTNVYADSFVIRGNEVSQIAPGQTGNIAD
BA.4         361    CVADYSVLYNFAPFFAFKCYGVSPTKLNDLCFTNVYADSFVIRGNEVSQIAPGQTGNIAD
XBB          361    CVADYSVIYNFAPFFAFKCYGVSPTKLNDLCFTNVYADSFVIRGNEVSQIAPGQTGNIAD
BA.2.86      361    CVADYSVLYNFAPFFAFKCYGVSPTKLNDLCFTNVYADSFVIKGNEVSQIAPGQTGNIAD
consensus    361    **********f*p*fa**************************r*n**s********n***

                    421......430.......440.......450.......460.......470.......480
                                       ++++++++++++++++++++++++++++RBM+++++++++
Wuhan-hu-1   421    YNYKLPDDFTGCVIAWNSNNLDSKVGGNYNYLYRLFRKSNLKPFERDISTEIYQAGSTPC
BA.1         421    YNYKLPDDFTGCVIAWNSNKLDSKVSGNYNYLYRLFRKSNLKPFERDISTEIYQAGNKPC
BA.2         421    YNYKLPDDFTGCVIAWNSNKLDSKVGGNYNYLYRLFRKSNLKPFERDISTEIYQAGNKPC
BA.4         421    YNYKLPDDFTGCVIAWNSNKLDSKVGGNYNYRYRLFRKSNLKPFERDISTEIYQAGNKPC
XBB          421    YNYKLPDDFTGCVIAWNSNKLDSKPSGNYNYLYRLFRKSKLKPFERDISTEIYQAGNKPC
BA.2.86      421    YNYKLPDDFTGCVIAWNSNKLDSKHSGNYDYWYRLFRKSKLKPFERDISTEIYQAGNKPC
consensus    421    *******************k****vg***n*l*******n****************nk**

                    481......490.......500.......510.......520.......530.......540
                    ++++++++++++++++++++++++++
Wuhan-hu-1   481    NGVEGFNCYFPLQSYGFQPTNGVGYQPYRVVVLSFELLHAPATVCGPKKSTNLVKNKCVNF
BA.1         481    NGVAGFNCYFPLRSYSFRPTYGVGHQPYRVVVLSFELLHAPATVCGPKKSTNLVKNKCVNF
BA.2         481    NGVAGFNCYFPLRSYGFRPTYGVGHQPYRVVVLSFELLHAPATVCGPKKSTNLVKNKCVNF
BA.4         481    NGVAGVNCYFPLQSYGFRPTYGVGHQPYRVVVLSFELLHAPATVCGPKKSTNLVKNKCVNF
XBB          481    NGVAGPNCYSPLQSYGFRPTYGVGHQPYRVVVLSFELLHAPATVCGPKKSTNLVKNKCVNF
BA.2.86      481    KG-KGPNCYFPLQSYGFRPTYGVGHQPYRVVVLSFELLHAPATVCGPKKSTNLVKNKCVNF
consensus    481    n*va*f***f**q**g*r**y***h************************************
```

SARS-CoV-2 Spike RBD (Arg319-Phe541)
Receptor Binding Domain at residue 438-506 (annotated with yellow +)

b

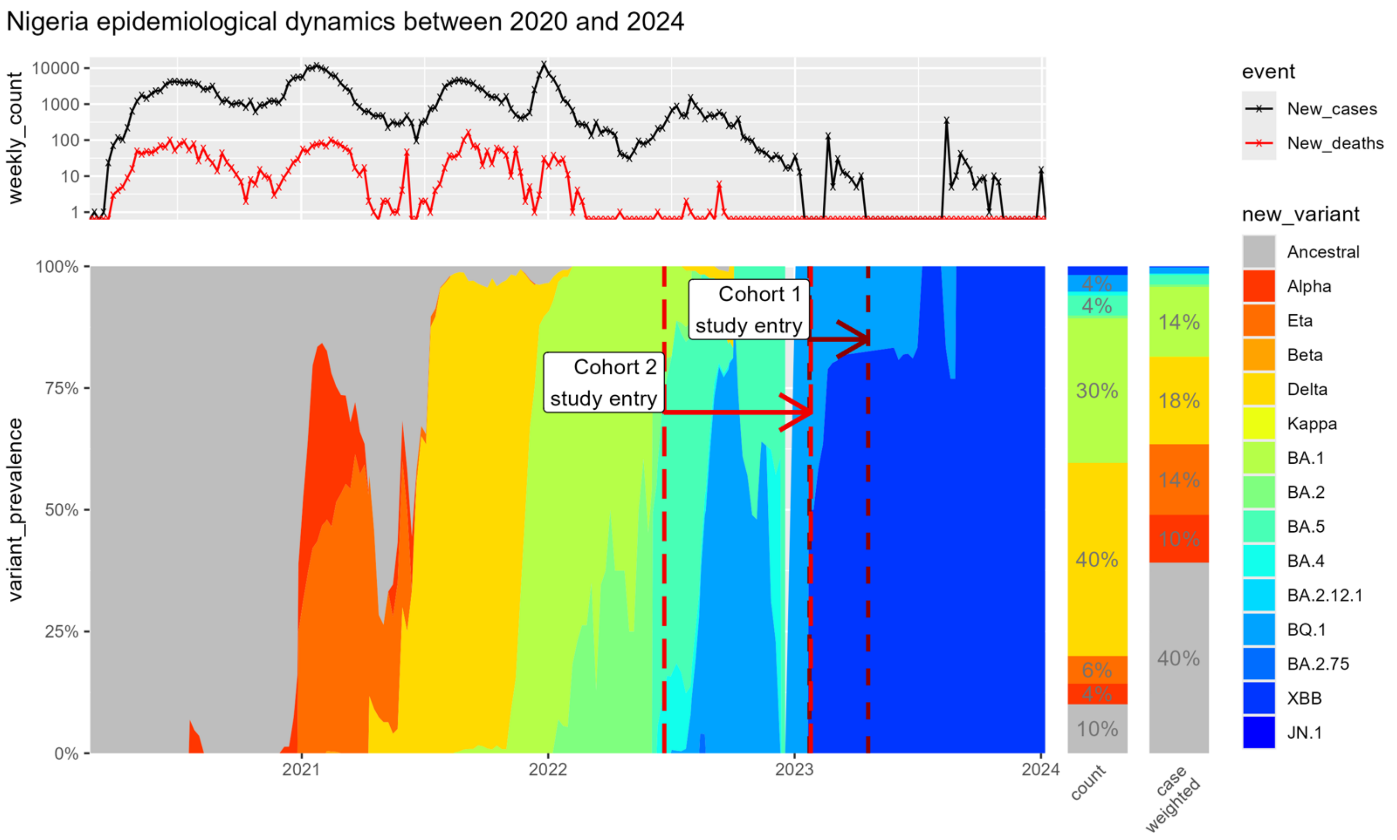

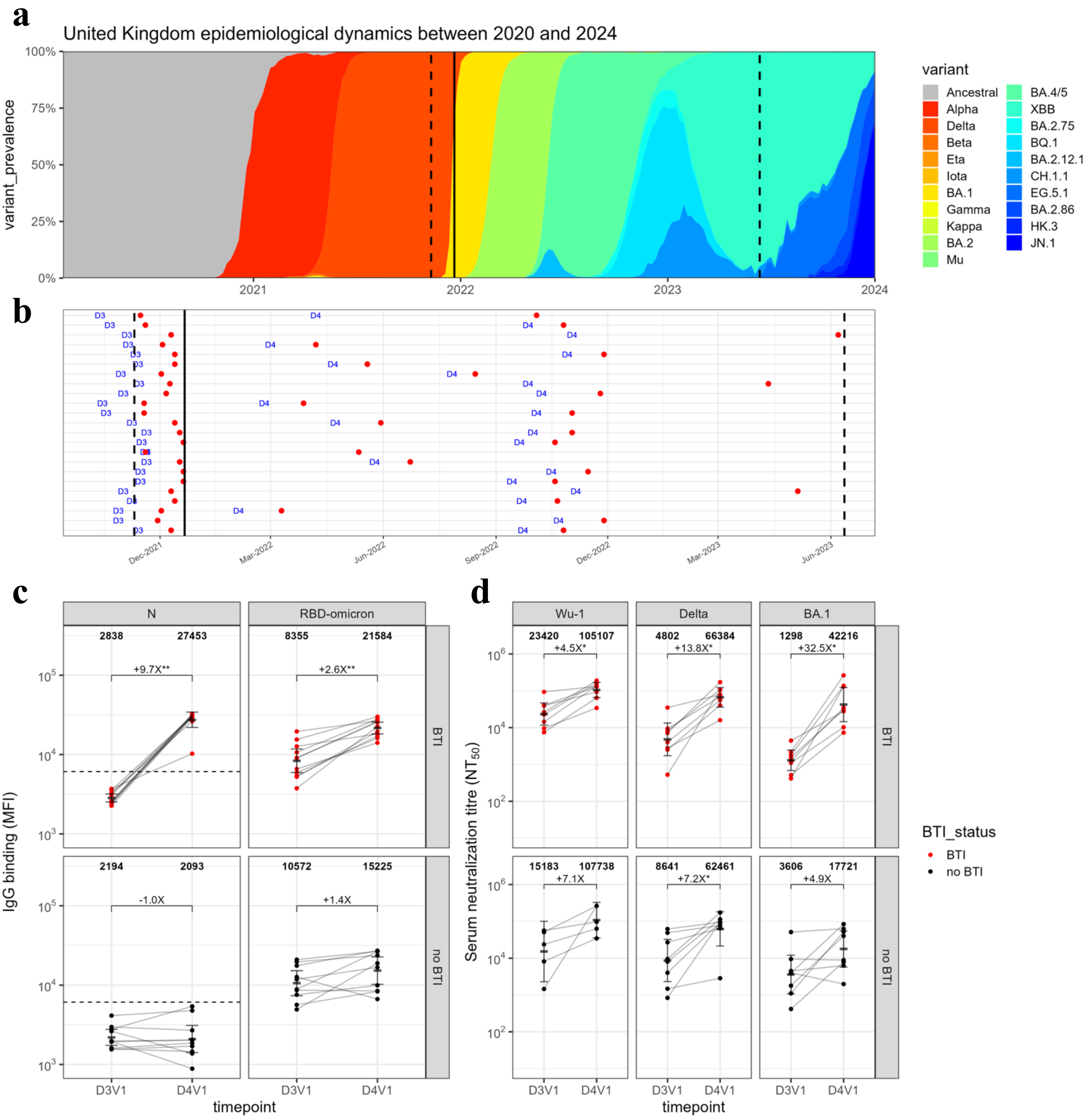
a
United Kingdom epidemiological dynamics between 2020 and 2024
variant_prevalence
100%
75%
50%
25%
0%
2021
2022
2023
2024
variant
Ancestral
Alpha
Delta
Beta
Eta
Iota
BA.1
Gamma
Kappa
BA.2
Mu
BA.4/5
XBB
BA.2.75
BQ.1
BA.2.12.1
CH.1.1
EG.5.1
BA.2.86
HK.3
JN.1
b
D3
D4
Dec-2021
Mar-2022
Jun-2022
Sep-2022
Dec-2022
Mar-2023
Jun-2023
c
N
RBD-omicron
2838
27453
+9.7X**
8355
21584
+2.6X**
BTI
2194
2093
-1.0X
10572
15225
+1.4X
no BTI
IgG binding (MFI)
D3V1
D4V1
timepoint
d
Wu-1
Delta
BA.1
23420
105107
+4.5X*
4802
66384
+13.8X*
1298
42216
+32.5X*
15183
107738
+7.1X
8641
62461
+7.2X*
3606
17721
+4.9X
Serum neutralization titre (NT50)
BTI_status
BTI
no BTI

a

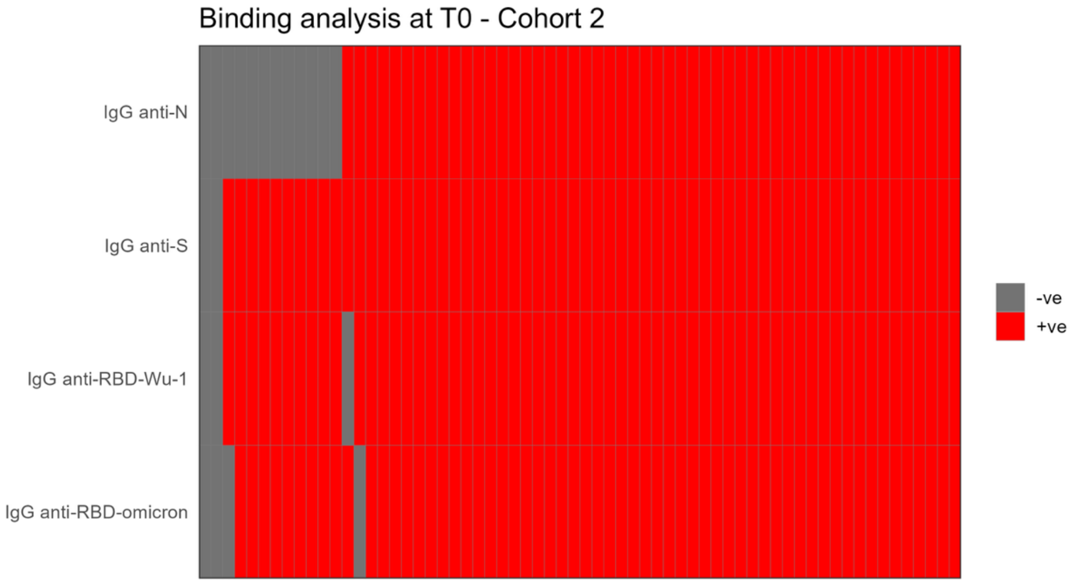
Binding analysis at T0 - Cohort 2
IgG anti-N
IgG anti-S
IgG anti-RBD-Wu-1
IgG anti-RBD-omicron
-ve
+ve

b

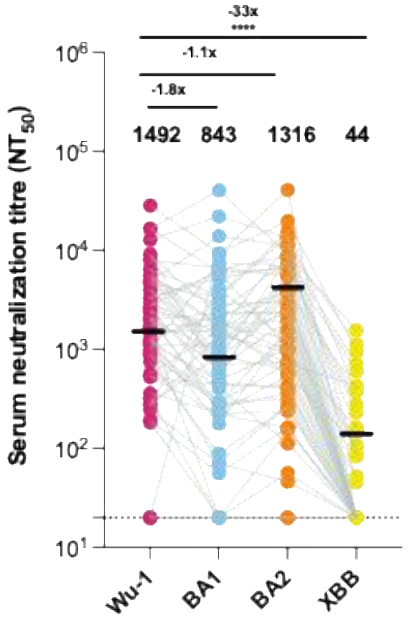
-33x
****
-1.1x
-1.8x
1492
843
1316
44
Serum neutralization titre (NT50)
Wu-1
BA1
BA2
XBB

T0

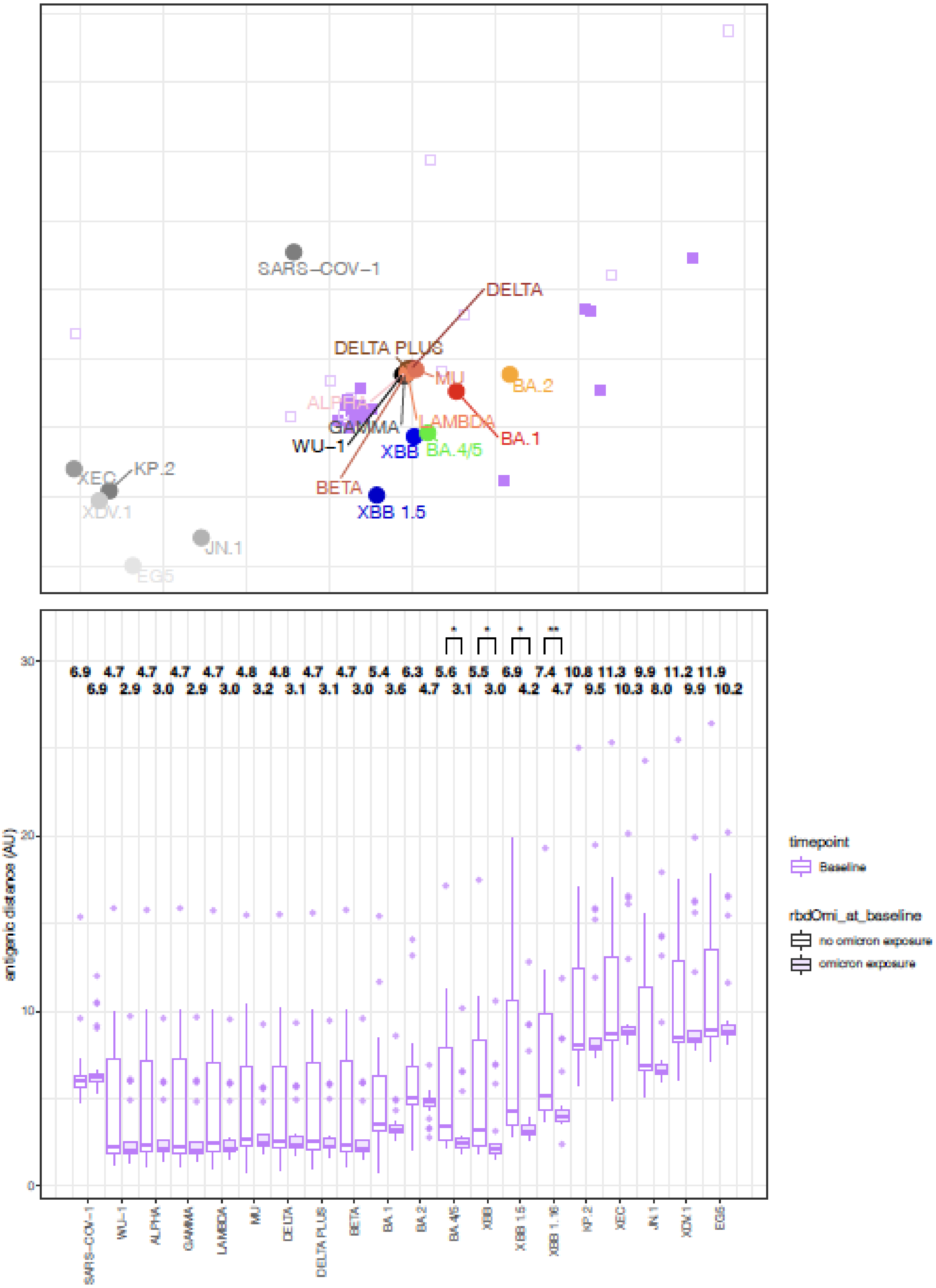
SARS−COV−1
DELTA
DELTA PLUS
MU
BA.2
ALPHA
LAMBDA
GAMMA
BA.1
WU−1
XBB
BA.4/5
XEC
KP.2
BETA
XDV.1
XBB 1.5
JN.1
EG5
6.9 4.7 4.7 4.7 4.7 4.8 4.8 4.7 4.7 5.4 6.3 5.6 5.5 6.9 7.4 10.8 11.3 9.9 11.2 11.9
6.9 2.9 3.0 2.9 3.0 3.2 3.1 3.1 3.0 3.6 4.7 3.1 3.0 4.2 4.7 9.5 10.3 8.0 9.9 10.2
antigenic distance (AU)
SARS−COV−1
WU−1
ALPHA
GAMMA
LAMBDA
MU
DELTA
DELTA PLUS
BETA
BA.1
BA.2
BA.4/5
XBB
XBB 1.5
XBB 1.16
KP.2
XEC
JN.1
XDV.1
EG5
timepoint
Baseline
rbdOmi_at_baseline
no omicron exposure
omicron exposure

a

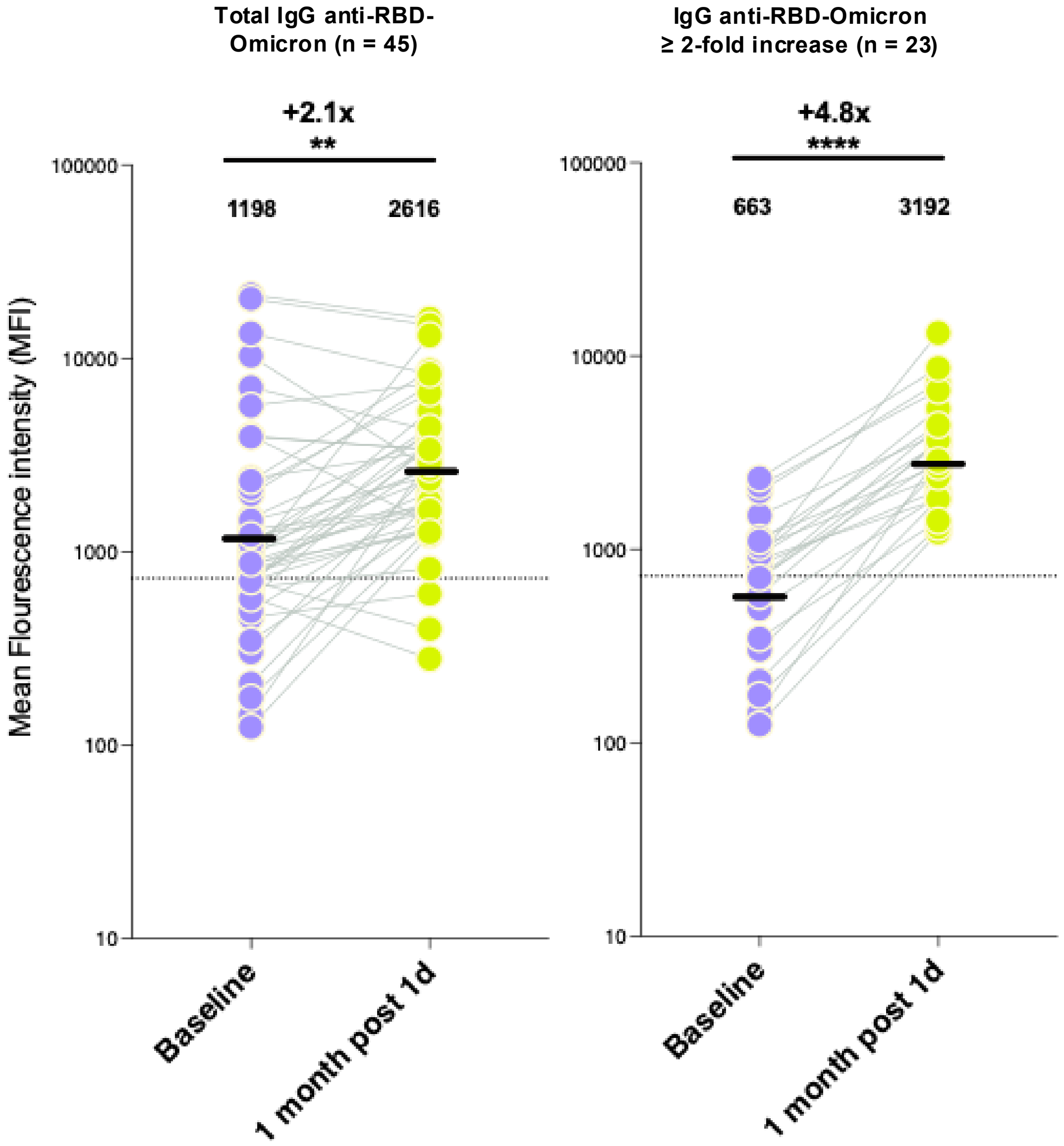

Total IgG anti-RBD-Omicron (n = 45)
+2.1x
**
1198
2616
Mean Flourescence intensity (MFI)
100000
10000
1000
100
10
Baseline
1 month post 1d
IgG anti-RBD-Omicron ≥ 2-fold increase (n = 23)
+4.8x
****
663
3192
100000
10000
1000
100
10
Baseline
1 month post 1d

a

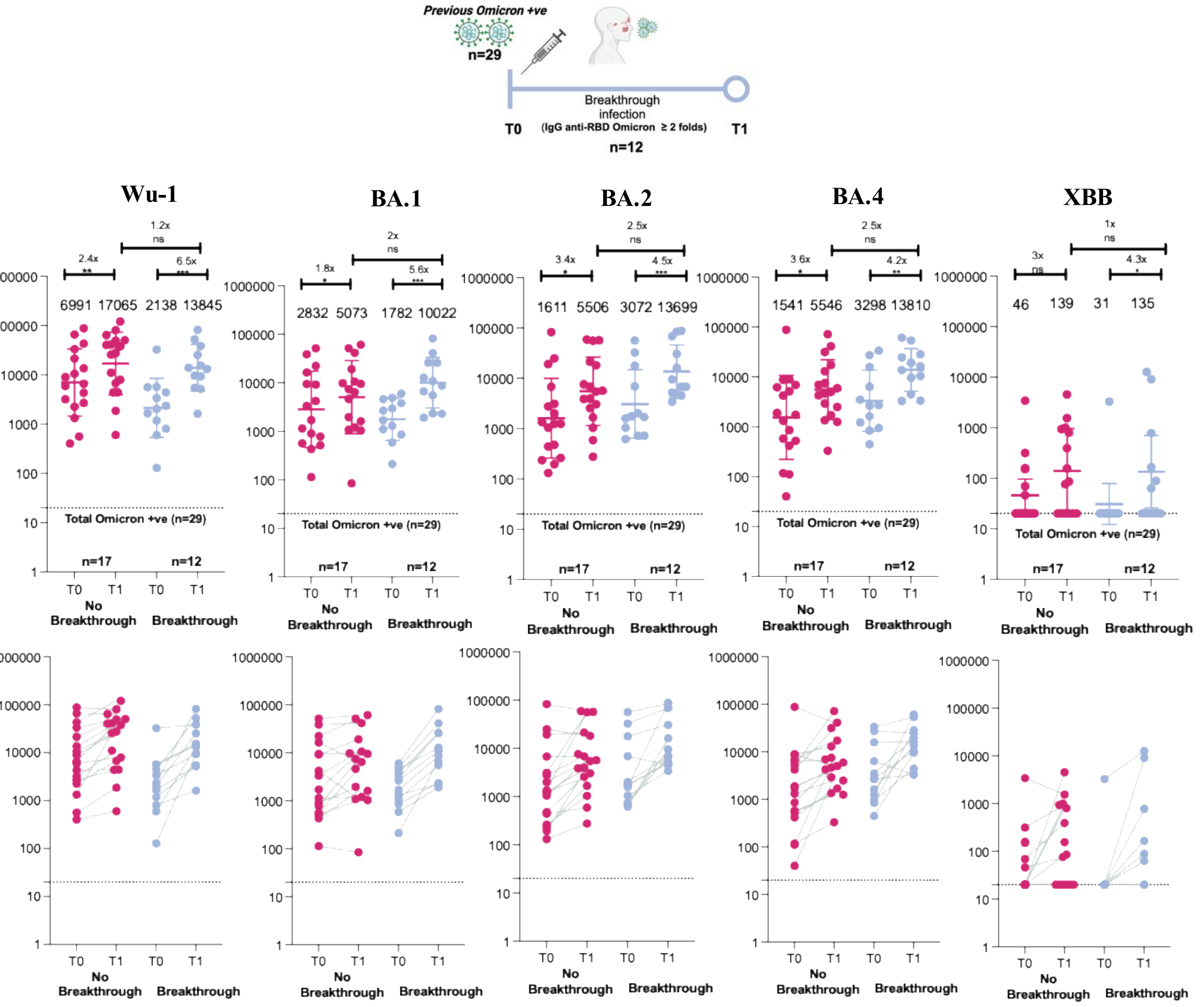
Previous Omicron +ve
n=29
Breakthrough infection (IgG anti-RBD Omicron ≥ 2 folds)
T0
T1
n=12
Wu-1
BA.1
BA.2
BA.4
XBB
Serum neutralization titre (NT50)
Total Omicron +ve (n=29)
n=17
n=12
No Breakthrough
Breakthrough
2.4x
1.2x
6.5x
6991 17065 2138 13845
1.8x
2x
5.6x
2832 5073 1782 10022
3.4x
2.5x
4.5x
1611 5506 3072 13699
3.6x
4.2x
1541 5546 3298 13810
3x
1x
4.3x
46 139 31 135

b

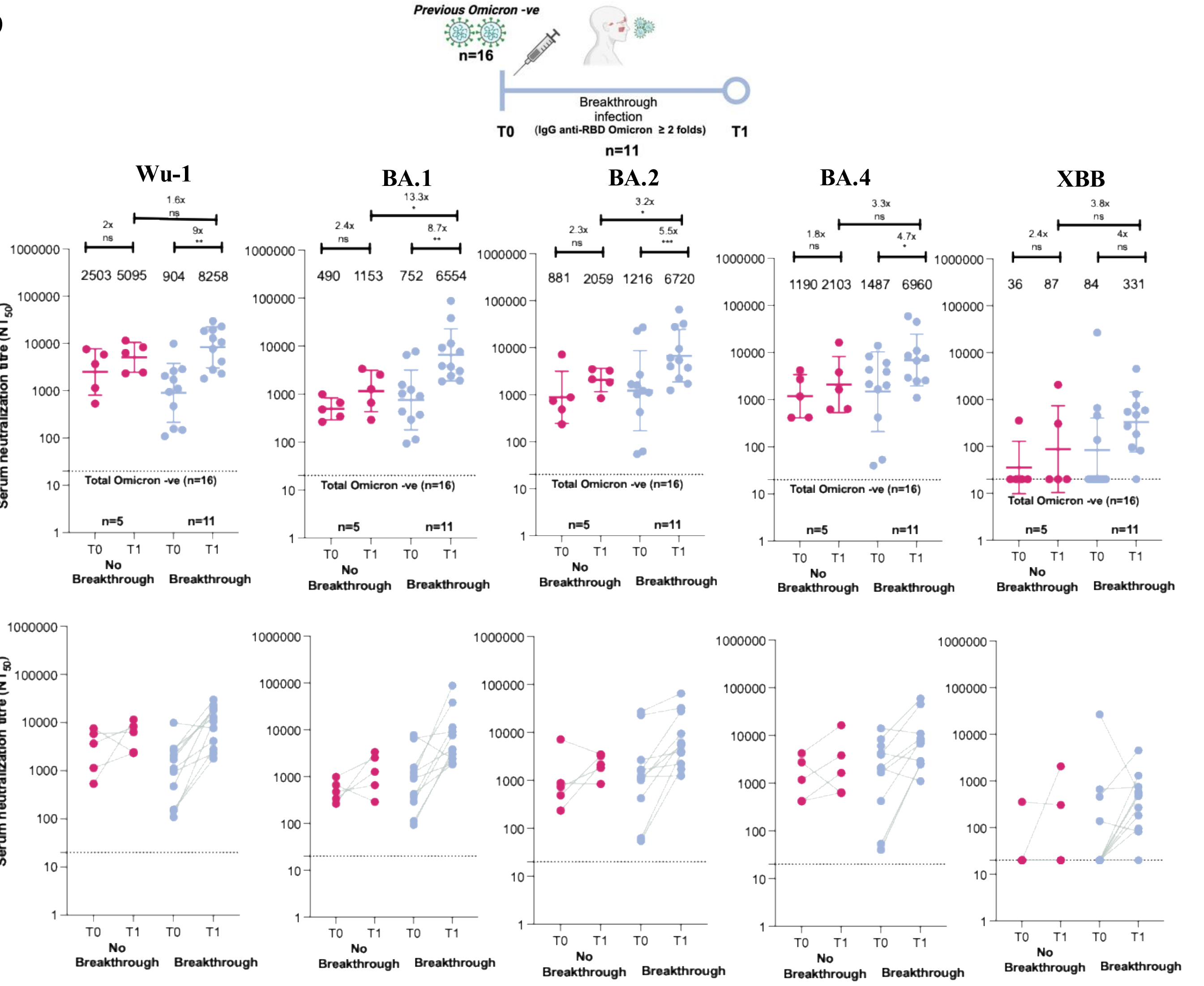
Previous Omicron -ve
n=16
Breakthrough infection (IgG anti-RBD Omicron ≥ 2 folds)
T0
T1
n=11
Wu-1
BA.1
BA.2
BA.4
XBB
Serum neutralization titre (NT50)
Total Omicron -ve (n=16)
n=5
n=11
No Breakthrough
Breakthrough
2x
1.6x
9x
2503 5095 904 8258
2.4x
13.3x
8.7x
490 1153 752 6554
2.3x
3.2x
5.5x
881 2059 1216 6720
1.8x
3.3x
4.7x
1190 2103 1487 6960
2.4x
3.8x
4x
36 87 84 331

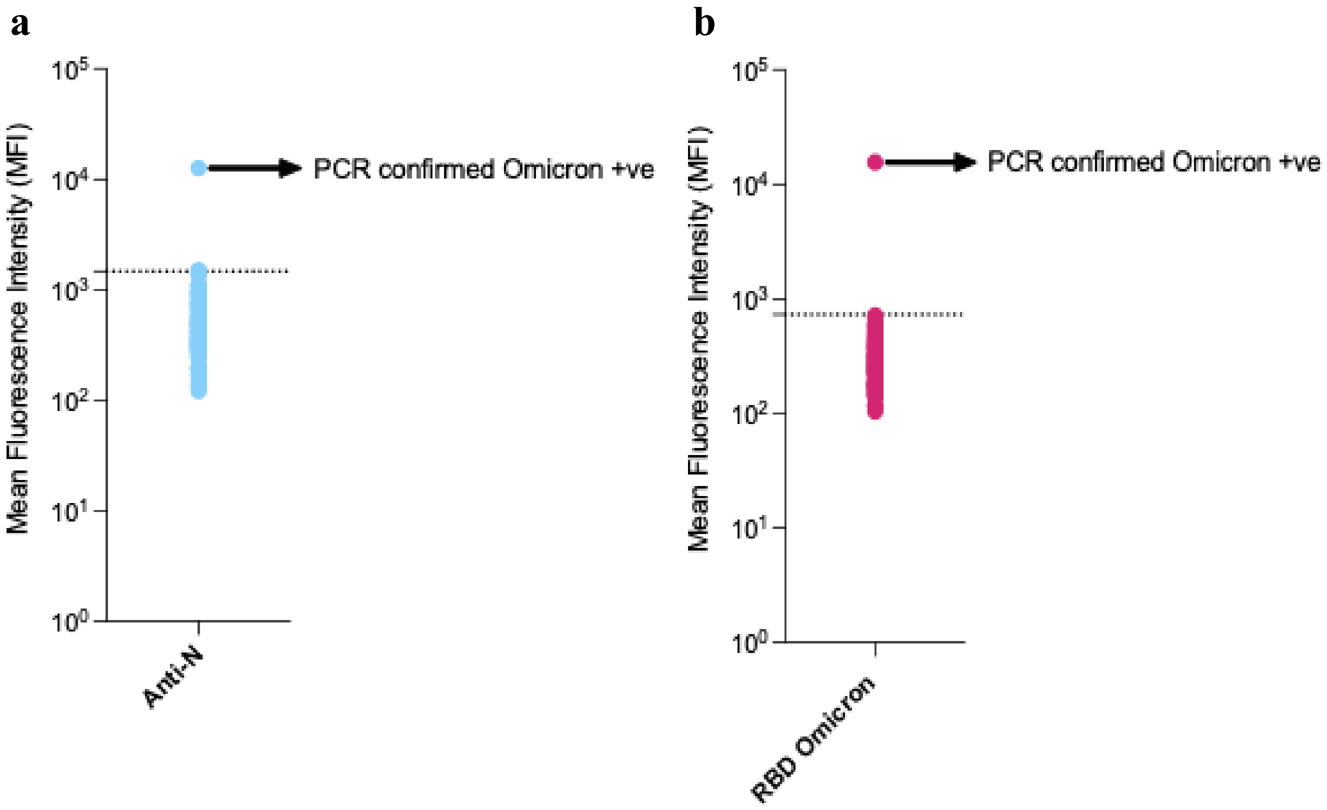
a
b
Mean Fluorescence Intensity (MFI)
PCR confirmed Omicron +ve
Anti-N
RBD Omicron

SUPPLEMENTAL INFORMATION

Document S1. Figures S1-S7, Tables S1 and S2.

Supplemental Data

Supplementary Figure 1: Spike Receptor Binding Domain alignment in variants of concern and population SARS-CoV-2 variant prevalence between 2020 and 2024. a) Multiple sequence alignment of SARS-CoV-2 Spike receptor binding domain (RBD). The sequence of SARS-CoV-2 Spike RBD from Wuhan-hu-1, BA.1, BA.2, BA.4, XBB, and BA2.86 were aligned by MAFFT, generated by boxshade, and annotated manually. Residue indexes are based on Wuhan-Hu-1, starting at Arg319. Residues different from consensus are in black on white background. Asterisks indicate completely conserved columns in the alignment. The receptor binding motif (RBM) residues are marked yellow plus signs (+). NCBI protein accession numbers are as follows: Wuhan-Hu-1 (NC_045512.2); BA.1 (OL672836.1); BA.2 (OM371884.1); BA.4 (ON373214.1); BA.2.86 (OR775659.1); XBB (XBB 1.5 OP790748.1 with S: P486S). **b)** SARS-CoV-2 epidemiological dynamics in Nigeria between 2020 and 2024. (top) The WHO reported weekly new cases (black) and new deaths (red) in Nigeria attributed to COVID-19. (bottom left) The weekly prevalence of each major variant on GISAID retrieved via the Outbreak.info API reported as a percentage. The stacked bar charts represented aggregated total proportion of sequences attributed to each variant based on sequence count (bottom middle) and based on sequence count weighted by the weekly number of new cases (bottom right).

Supplementary Figure 2: Epidemiological context, vaccination timeline, and validation of BA.1 Omicron-specific antibody and neutralization assays using a UK cohort. a) SARS-CoV-2 epidemiological dynamics in the United Kingdom between 2020 and 2024. **b)** The vaccination history for the UK NBR118 cohort. The blue text represents their vaccination dose and timing, whilst the red dot represents when the samples were collected. The left dotted line represents the first post-dose 3 sample (2021-11-10), the center solid line represents the last post-dose 3 sample (2021-12-21), and the right dotted line represents the last post-dose 4 sample (2023-06-12). **c)** Total IgG of anti-nucleocapsid (N) and anti-Receptor Binding Domain Omicron-specific (RBD-Omicron) antibodies and **d)** pseudotyped virus neutralization titers of Wu-1, Delta, and BA.1 in the UK NBR118 cohort. Data are representative of two independent experiments comprised of two technical replicates. Geometric mean titer (GMT) of neutralizing antibody responses is shown numerically above each timepoint and as horizontal black lines. Data points were compared using Holm-corrected two-tailed Wilcoxon test. *$P < 0.05$; **$P < 0.01$; ***$P < 0.001$; ****$P < 0.0001$; no * = not significant. Dashed lines indicate limit of neutralization response detection. Fold changes are represented above the corresponding horizontal comparative lines. Breakthrough infection (BTI) is defined by a greater than 2-fold increase in total anti-nucleocapsid IgG binding, with n=11 with BTI and n=10 without BTI.

Supplementary Figure 3: Pre-vaccination antibody response in participants from a 2023 vaccine trial in Nigeria with Omicron exposure defined by IgG anti-RBD-Omicron status (Cohort 2, n = 58). a) Participants recruited with neutralization response data prior to vaccination (T0) who were tested for total IgG antibodies against SARS-CoV-2 Wildtype (Wu-1) anti-Nucleocapsid (anti-N), anti-Spike (S), anti-Wu-1 Receptor Binding Domain (anti-RBD-Wu-1), and anti-Omicron BA.1 RBD (anti-RBD-Omicron). **b)** Pseudotyped virus neutralization titers of Wu-1, BA.1, BA.2 and XBB viruses in (n = 58) participants in cohort 2. Data are representative of two independent experiments comprised of two technical replicates. Geometric mean titer (GMT) of neutralizing antibody responses is shown numerically above each timepoint and as horizontal black lines. Data points were compared and assessed for significance using two-tailed Wilcoxon signed-rank tests. *$P < 0.05$; **$P < 0.01$; ***$P < 0.001$; ****$P < 0.0001$; no * = not significant. Dashed lines indicate limit of neutralization response detection. Fold changes are represented above the corresponding horizontal comparative lines.

Supplementary Figure 4: Antigenic map and antigenic distances of pre-vaccination neutralization responses stratified by Omicron exposure. (Top) Antigenic map of Pre-vaccination (T0) surrogate viral neutralization test titers against the receptor binding domain (RBD) of SARS-CoV-1 and a series of SARS-CoV-2 variants of concerns, represented by coloured circles. Squares represent individual, where filled squares represent patients with omicron exposure and empty squares represent patients with no omicron exposure at study entry. (Bottom) The antigenic distance between different RBD targets and patients grouped by omicron exposure at study entry. Mean of antigenic distance is shown above each RBD target (paired values - no omicron exposure is on the top left and omicron exposed antigenic distance is on the bottom right for each antigen pair). The boxplot represents the middle line represents the median, the hinges 25th and 75th percentiles, and whiskers are 1.5 x IQR from the hinge. Data points were compared using Friedman's Test with Holm-corrected post-Hoc Wilcoxon test. *$P < 0.05$; **$P < 0.01$; ***$P < 0.001$; ****$P < 0.0001$; absence of any (*) = not significant.

Supplementary Figure 5: Kinetics of IgG binding antibody responses against SARS-CoV-2 Omicron RBD. a) Total IgG of anti-Receptor Binding Domain (RBD) Omicron-specific antibodies before and after one dose of the Ad26.COV2.S vaccine in (n = 45) participants (left panel). Participants who had ≥ 2-fold increase in anti-RBD-Omicron titers from pre-vaccination to 1-month post-dose 1 (n = 23) are shown (right panel). Geometric mean of the Mean Fluorescence Intensity (MFI) from the Luminex binding antibody assay is shown numerically above each timepoint and as black horizontal lines.

Supplementary Figure 6: Plasma neutralization responses stratified by pre-vaccination Omicron exposure status with or without breakthrough infection after one dose of Ad26.COV2.S vaccine. Pseudotyped virus neutralization titers against Wildtype (Wu-1), BA.1, BA.2, BA.4 and XBB across two consecutive time points: T0 (prior to first-dose vaccination) and T1 (1 month after first-dose vaccination). Titers in **a)** pre-vaccination Omicron-exposed participants (n = 29) stratified by Omicron breakthrough infection (BTI, n = 12) and without BTI (n = 17), and in **b)** pre-vaccination Omicron-unexposed participants (n = 16) stratified by Omicron BTI (n = 11) and without BTI (n = 5). Data are representative of two independent experiments comprised of two technical replicates. Geometric mean titer (GMT) of antibodies is shown numerically above each timepoint. Data points were compared using two-tailed Wilcoxon signed-rank tests and shown as GMT with 95% CI. *$P < 0.05$; **$P < 0.01$; ***$P < 0.001$; ****$P < 0.0001$; ns = not significant.

Supplementary Figure 7: Validation of Luminex assay for quantification of binding antibodies using pre-pandemic sera from (n = 93) participants. a) Binding antibodies against Wu-1 anti-Nucleocapsid (anti-N) and **b)** binding antibodies against Omicron Receptor Binding Domain (anti-RBD-Omicron) in (n = 93) pre-pandemic control sera samples. Data are represented as Mean Fluorescence Intensity (MFI). Cut-offs were determined using Mean + 3 standard deviations of the mean.

Supplementary Table 1: Baseline characteristics of study participants in Cohort 2

**Characteristic**	
Total number, n(%)	64 (100)
Age, median years (IQR)	37 (25, 46)
Female sex, n(%)	32 (50)
IgG anti-N, n(%)	52 (81)

Supplementary Table 2: Baseline characteristics of participants depleted for Wu-1

Characteristic	Total	Omicron exposure	No Omicron exposure
Total number, n(%)	29 (100)	21 (70)	8 (30)
Age, median years (IQR)	36 (28, 41)	33 (26, 41)	36 (30, 41)
Female sex, n(%)	17 (59)	11 (65)	6 (35)
IgG anti-N, n(%)	28 (97)	21 (75)	6 (21)
